# Refined spatial temporal epigenomic profiling reveals intrinsic connection between PRDM9-mediated H3K4me3 and the fate of double-stranded breaks

**DOI:** 10.1038/s41422-020-0281-1

**Published:** 2020-02-11

**Authors:** Yao Chen, Ruitu Lyu, Bowen Rong, Yuxuan Zheng, Zhen Lin, Ruofei Dai, Xi Zhang, Nannan Xie, Siqing Wang, Fuchou Tang, Fei Lan, Ming-Han Tong

**Affiliations:** 1 State Key Laboratory of Molecular Biology, Shanghai Key Laboratory of Molecular Andrology, CAS Center for Excellence in Molecular Cell Science, Shanghai Institute of Biochemistry and Cell Biology, Chinese Academy of Sciences, University of Chinese Academy of Sciences, Shanghai, 200031 China; 2 Shanghai Key Laboratory of Medical Epigenetics, International Co-laboratory of Medical Epigenetics and Metabolism, Ministry of Science and Technology, Institutes of Biomedical Sciences, Fudan University, and Key Laboratory of Carcinogenesis and Cancer Invasion, Ministry of Education, Liver Cancer Institute, Zhongshan Hospital, Fudan University, Shanghai, 200032 China; 3 Beijing Advanced Innovation Center for Genomics, Biomedical Institute for Pioneering Investigation via Convergence, College of Life Sciences, Peking University, Beijing, 100871 China; 4 Ministry of Education Key Laboratory of Cell Proliferation and Differentiation, Beijing, 100871 China; 5 Peking-Tsinghua Center for Life Sciences, Peking University, Beijing, 100871 China

**Keywords:** DNA recombination, Histone post-translational modifications

## Abstract

Meiotic recombination is initiated by the formation of double-strand breaks (DSBs), which are repaired as either crossovers (COs) or noncrossovers (NCOs). In most mammals, PRDM9-mediated H3K4me3 controls the nonrandom distribution of DSBs; however, both the timing and mechanism of DSB fate control remain largely undetermined. Here, we generated comprehensive epigenomic profiles of synchronized mouse spermatogenic cells during meiotic prophase I, revealing spatiotemporal and functional relationships between epigenetic factors and meiotic recombination. We find that PRDM9-mediated H3K4me3 at DSB hotspots, coinciding with H3K27ac and H3K36me3, is intimately connected with the fate of the DSB. Our data suggest that the fate decision is likely made at the time of DSB formation: earlier formed DSBs occupy more open chromatins and are much more competent to proceed to a CO fate. Our work highlights an intrinsic connection between PRDM9-mediated H3K4me3 and the fate decision of DSBs, and provides new insight into the control of CO homeostasis.

## Introduction

Meiosis is a specialized form of cell division that generates haploid gametes from diploid cells, and is essential for sexual reproduction and evolution^1,2^. Fundamental to meiosis is the process of meiotic recombination, which leads to the transmission of new combinations of linked alleles to the next generation^1,2^. Meiotic recombination is initiated by the introduction of programmed DNA double-strand breaks (DSBs) by the topoisomerase-like transesterases, SPO11 and TOPVIBL^1,3–6^. Following DSB formation, single-stranded DNA (ssDNA) ends are engaged in the process of repair, which results in the loading of RAD51 and DMC1^1,2,7^. This facilitates the search for homologous chromosomes (homologs) in the majority of strand invasion and the formation of single-end invasion strand exchange intermediates (SEIs)^2,8–10^. SEIs can be resolved either by synthesis-dependent strand annealing (SDSA) to generate only noncrossover (NCO) recombinants, or by double Holliday junctions (dHJs) to generate crossover (CO)/NCO recombinants^2,8–19^. Only a small fraction of DSBs are subsequently repaired to produce COs, whereas the remaining ones lead to the formation of NCOs^2,8^. DSBs occur most often at preferred sites termed hotspots, whose locations become marked by H3K4me3; in most mammals, this is determined by the histone methyltransferase PRDM9^20–24^. Despite recent advances, much remains to be known about the dynamic nature and function of critical epigenetic factors involved in the recombination process and in particular, in the DSB fate decision.

## Results

### Data generation and validation

**Fig. 1:**
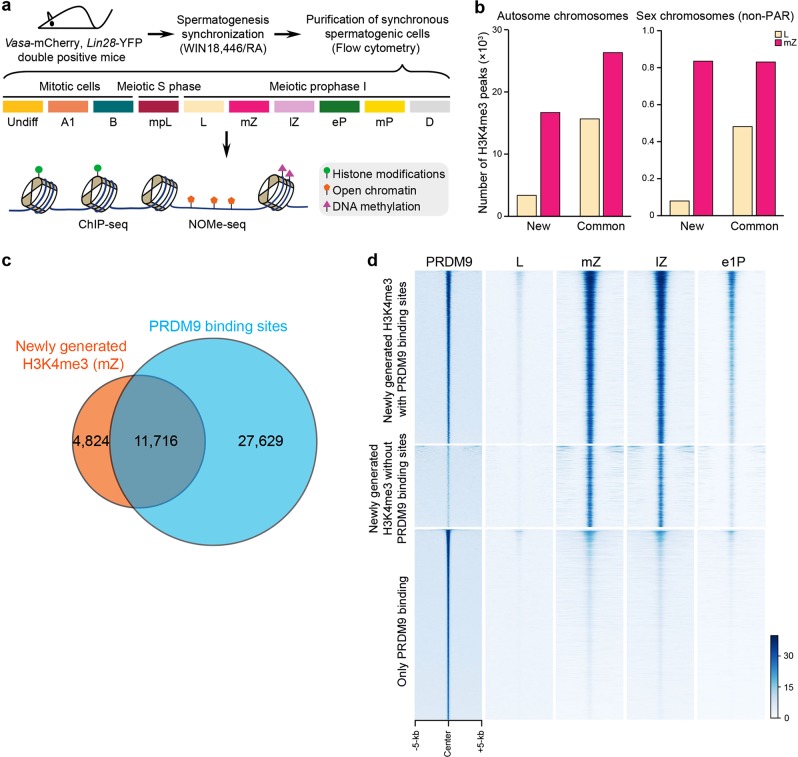
Identification of PRDM9-dependent H3K4me3 modifications in the mouse genome. **a** Schematic workflow. Male mice carrying both *Vasa*-mCherry and *Lin28*-YFP alleles were treated with WIN 18,446/retinoic acid to synchronize spermatogenesis. Testes were then digested and the synchronous spermatogenic cells were sorted by flow cytometry. In total, spermatogenic cells at ten stages, including mitotic cells (Undiff undifferentiated spermatogonia, A1 type A1 spermatogonia, B type B spermatogonia), meiotic S phase cells (pL preleptotene spermatocytes) and meiotic prophase I cells (L leptotene spermatocytes, mZ mid-zygotene spermatocytes, lZ late-zygotene spermatocytes, eP early-pachytene spermatocytes, mP mid-pachytene spermatocytes, D diplotene spermatocytes) were collected for ChIP-seq and NOMe-seq analyses. **b** Numbers of the newly generated (new) and common (common) H3K4me3 peaks in leptotene (L) and mid-zygotene (mZ) spermatocytes. **c** Venn diagram showing the overlap of the newly generated H3K4me3 peaks in mid-zygotene with PRDM9-binding sites (PRDM9 affinity-seq data). **d** Heatmap of H3K4me3 normalized tag density on PRDM9-binding sites. Each row represents a PRDM9-binding site of ± 5 kb around the center and ranked by PRMD9 tag density from the highest to the lowest. H3K4me3 and PRDM9 tag densities were calculated using H3K4me3 ChIP-seq reads or PRDM9 affinity-seq reads with 50-bp resolution. PRDM9 PRDM9-binding sites, e1P early 1-pachytene.

To address whether and how epigenetic programs direct meiotic recombination, we used an approach to purify homogeneous populations of all subtypes (meiotic stages) of mouse spermatogenic cells^25^, and assayed their epigenomes. We applied ChIP-seq (chromatin immunoprecipitation (ChIP) with sequencing) to spermatogenic cells for seven critical histone modifications, H3K4me3, H3K9me2, H3K9me3, H3K27me3, H3K36me3, H3K4me1 and H3K27ac, and NOMe-seq (Nucleosome Occupancy and Methylome Sequencing) for the chromatin state and nucleosome positioning for each sample with two biological replicates (Fig. 1a; Supplementary information, Fig. S1 and Table S1). We also performed ChIP-seq and NOMe-seq on synchronous leptotene and zygotene spermatocytes from *Prdm9*^−/−^, *Spo11*^−/−^, and *Dmc1*^−/−^ mice (PRDM9 and SPO11 are required for generation of DSBs during meiosis; DMC1 facilitates the search for homologs) (Supplementary information, Table S1). We generated about 18.5 M (18,389,757) unique reads for each ChIP-seq (Supplementary information, Table S1), and confirmed the high reproducibility of our datasets (Pearson’s correlation coefficient > 0.8, between the two replicates) (Supplementary information, Table S2). We performed integrative analyses using our epigenome data and publicly available SPO11-oligo and DMC1-ssDNA profiles representing recombination hotspots^20,26^.

### Identification of PRDM9-mediated H3K4me3

It is well-known that PRDM9 and PRDM9-dependent H3K4me3 play critical roles in positioning DSB hotspots, but previous data were obtained from mixed aggregates of testicular cells^20,26–28^, thus lacking spatiotemporal information in meiotic prophase I spermatocytes. Our analyses allowed us to identify PRDM9-dependent H3K4me3 by comparing the H3K4me3 profiles between the neighboring stages of homogeneous spermatogenic cells. Through this strategy, a total of 3358 and 16,540 de novo H3K4me3 peaks were identified in leptotene and mid-zygotene spermatocytes, respectively (Fig. 1b; Supplementary information, Table S3). We also identified 15,614 and 26,225 common (present in any of the prior spermatogonia stages) H3K4me3 peaks in leptotene and mid-zygotene spermatocytes, respectively (Fig. 1b and Supplementary information, Table S4; the sex chromosomes are excluded from subsequent analyses since the role of PRDM9 on them is different from on autosomes). The newly generated H3K4me3 peaks were mainly localized to intergenic (47.0%) and intronic (37.0%) regions, whereas the common H3K4me3 peaks were mainly enriched in promoters (63.2%) and likely transcriptionally related (Supplementary information, Fig. S2a). The average strength (tag density) of the common H3K4me3 peaks was much stronger than the newly generated ones, but comparable to the strongest newly generated ones (Supplementary information, Fig. S2b, c). Of note, although ChIP-seq signals reflect population averages, the comparable H3K4me3 signals between the strongest newly generated peaks and the nearby strong promoter ones (Supplementary information, Fig. S2c), indicate that they are probably fully methylated in all cells. Importantly, most DSB hotspots (75.7% defined by SPO11-oligo; 84.5% defined by DMC1-ssDNA; hereafter DSB hotspots are defined by SPO11-oligo unless explicitly mentioned, see “Materials and Methods” section for more details) overlapped with the newly generated H3K4me3 peaks, whereas very few hotspots overlapped with the common H3K4me3 peaks (5.9% defined by SPO11-oligo; 6.6% defined by DMC1-ssDNA) (Supplementary information, Fig. S2d, e). We also observed a strong positive correlation between DSB hotspot strength and H3K4me3 signal (Supplementary information, Fig. S2f, g)^20,26^. Of the minority of DSB hotspots (24.3%) that were scored as absent for H3K4me3, many were still decorated by weak H3K4me3 signals, which were below our stringent detection threshold to avoid increase of the probability of false positivity (Supplementary information, Fig. S2h). Significant enrichment of the PRDM9 DNA-binding motif was found in the vicinity of the newly generated H3K4me3 but not the common H3K4me3 regions (Supplementary information, Fig. S3a, b)^20,26,27^. Consistently, 11,716 (70.8%) of 16,540 newly generated H3K4me3 marks overlap with PRDM9-binding sites defined by the PRDM9-affinity-Seq^29^ (Fig. 1c, d). Interestingly, based on PPDM9 and H3K4me3 track, we identified a total of 4839 sites, 302 of which are co-modified by H3K27ac and H3K36me3, not reported as SPO11-oligo positive^26^. We found that these sites are associated with very weak SPO11-oligo signals, which might not pass the threshold of the analyses, but may be potential DSB sites considering the epigenome information (Supplementary information, Fig. S4a, b). These results indicate that the newly generated H3K4me3 are introduced by the stage-specifically expressed methyltransferase, PRDM9. Previous data have demonstrated that PRDM9 expression starts in type B spermatogonia, reaches its peak in the mid-zygotene stage, and disappears in late-zygotene and early-pachytene^30,31^, which we confirmed by nuclear spreading staining (Supplementary information, Fig. S5).

To directly test the role of PRDM9 in formation of the new H3K4me3 signals, we analyzed H3K4me3 marks and DSB hotspots (defined by DMC1-ssDNA) in synchronized spermatogenic cells from the *Prdm9*^−/−^ mice, and compared DSB hotspots in the *Prdm9*^−/−^mice with H3K4me3 in the wild-type (WT) mice. Of all the DSB hotspots in the *Prdm9*^−/−^ mice, 71.6% overlapped with H3K4me3 in *Prdm9*^−/−^ spermatocytes, with the vast majority (74.0%) coinciding with common H3K4me3 marks in WT spermatocytes, whereas essentially none (1.8%) overlapped with the newly generated H3K4me3 signals in WT spermatocytes (Supplementary information, Fig. S2c, i). In contrast, similar profiles of newly generated H3K4me3 were observed in *Spo11*^−/−^ and *Dmc1*^−/−^ mice, compared with WT mice, indicating that these H3K4me3 signals are independent of DSB formation and/or repair (Supplementary information, Fig. S6a, b) as previously reported^20^. Thus, production of the majority of newly generated H3K4me3 in WT mice is catalyzed by PRDM9 (henceforth referred to as PRDM9-mediated H3K4me3), and therefore PRDM9-mediated H3K4me3 could serve as a predictor for recombination hotspots in WT mice.

### Dynamics and regulation of PRDM9-mediated H3K4me3 signals during meiotic recombination

**Fig. 2:**
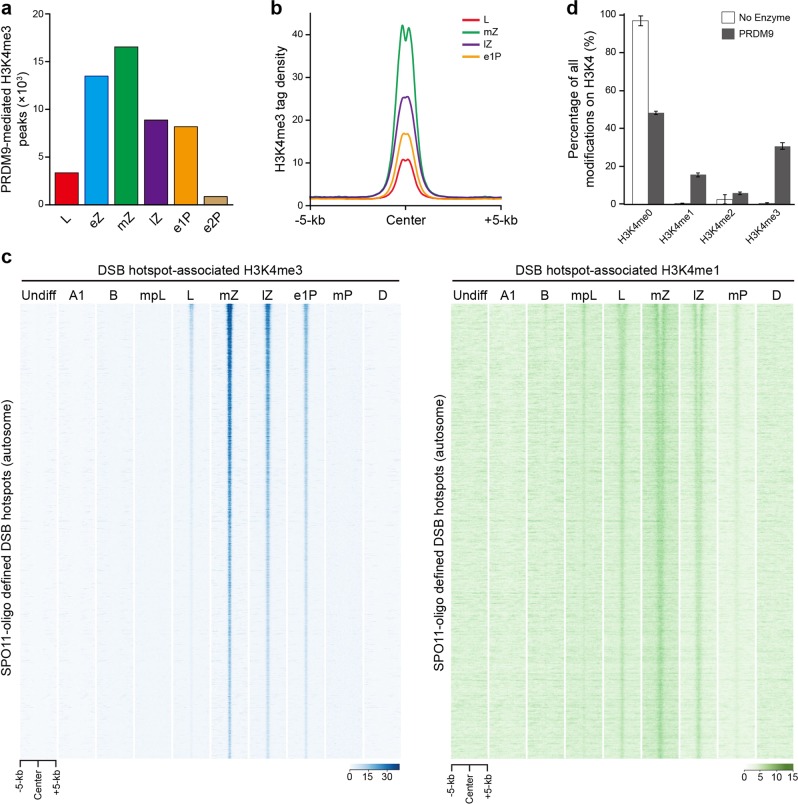
The dynamics of PRDM9-mediated H3K4me3 marks during progression of recombination. **a** Numbers of PRDM9-mediated H3K4me3 peaks in leptotene (L), early-zygotene (eZ), mid-zygotene (mZ), late-zygotene (lZ), early 1-pachytene (e1P), early 2-pachytene (e2P), and mid-pachytene (mP) spermatocytes. **b** Profile of the averaged H3K4me3 tag density on early-forming hotspot-associated H3K4me3 peaks in leptotene, mid-zygotene, late-zygotene, and early 1-pachytene. H3K4me3 tag density was calculated using H3K4me3 ChIP-seq read coverage with 50-bp resolution. **c** Heatmaps of H3K4me3 (left) and H3K4me1 (right) normalized tag density on hotspot-associated H3K4me3 peaks in premeiotic and meiotic cells. Each row represents a hotspot-associated H3K4me3 peak of ±5 kb around the peak center and ranked based on SPO11-oligo density from the highest to the lowest. **d** Histone Methyltransferase Assay using recombinant Prdm9 and oligonucleosomes. The percentage of each methylation state on H3K4 site was quantified by histone mass spectrometry. The blank box represents no enzyme control. Undiff undifferentiated spermatogonia, A1 type A1 spermatogonia, B type B spermatogonia, mpL mid-preleptotene spermatocytes, L leptotene spermatocytes, mZ mid-zygotene spermatocytes, lZ late-zygotene spermatocytes, e1P early 1-pachytene spermatocytes, mP mid-pachytene spermatocytes, D diplotene spermatocytes.

To determine the dynamics of PRDM9-mediated H3K4me3 marks during meiotic recombination, we first examined the distribution of PRDM9-mediated H3K4me3 marks in different substages of leptotene, zygotene, and pachytene in detail. PRDM9-mediated H3K4me3 modifications began to appear in leptotene (3358 peaks), increased their peak numbers in early-zygotene (13,490), reached their peak levels in mid-zygotene (16,540), partially persisted in late-zygotene (8880) and early 1-pachytene (collected 240 h after retinoic acid treatment during spermatogenesis synchronization) (8187), and disappeared in early 2-pachytene (collected 276 h after retinoic acid treatment during spermatogenesis synchronization) (883) and mid-pachytene (749) (Figs. 1b and 2a). In line with this, we identified 2773 (leptotene), 9440 (early-zygotene), 10,137 (mid-zygotene), 7681 (late-zygotene), 7375 (early 1-pachytene), and 11 (early 2-pachytene) hotspot-associated H3K4me3 peaks, which are defined by PRDM9-mediated H3K4me3 marks overlapping with the DSB hotspots, respectively, from the above six subtypes of spermatocytes (Supplementary information, Fig. S8a). We found that the number of hotspot-associated H3K4me3 peaks was much less in leptotene (2773) and early-zygotene (9440) than mid-zygotene (10,137) (Supplementary information, Fig. S8a). Recent study by Camerini-Otero and colleagues also found that the H3K4me3 marks appear in leptotene and are maximal in zygotene^32^. Thus, these data led us to propose that the majority of DSBs might occur at mid-zygotene. This hypothesis is inconsistent with earlier suggestions that DSBs mainly form at leptotene, but in agreement with the peak of DMC1 and RAD51 foci at zygotene^33^. It will be of interest to further test this hypothesis in the future. 17.4% and 38.7% of PRDM9-mediated H3K4me3 were not associated with DSB hotspots in leptotene and zygotene, respectively, indicating that they did not drive DSB formation. However, the strength of these non-hotspot H3K4me3 modifications was weaker compared with the hotspot-associated H3K4me3 marks, see discussion (Supplementary information, Fig. S7a, b). While the majority of hotspot-associated H3K4me3 marks emerged in the mid-zygotene stage, there are still 7681 and 7375 such peaks in late-zygotene and early 1-pachytene, respectively (Supplementary information, Fig. S8a). Since cells in late-zygotene and onwards did not express PRDM9 (Supplementary information, Fig. S5), hotspot-associated H3K4me3 marks in those spermatocytes are likely evolved and maintained from the previous stages such as mid-zygotene. Strikingly, nearly all (94.6%; 2623) hotspot-associated H3K4me3 modifications in leptotene (hereafter referred to as early-forming H3K4me3) persisted through mid-late zygotene to early 1-pachytene (Supplementary information, Fig. S8a). To determine whether and how the early-forming H3K4me3 marks change with recombinational progression, we compared their widths and strengths among leptotene, mid-zygotene, late-zygotene, and early 1-pachytene. We observed that the width and strength of the early-forming H3K4me3 underwent a drastic increase in mid-zygotene, followed by a gradual decrease in late-zygotene and early 1-pachytene (Fig. 2b; Supplementary information, Figs. S2c, S8b–e). The increase of H3K4me3 width and strength from leptotene to mid-zygotene is thought to reflect that the newly formed H3K4me3 modifications are generated near original ones at the same loci or its homolog loci in the same nuclei, although we cannot exclude the possibility that the new ones are generated at the same loci in different nuclei; in contrast, the decline of H3K4me3 width and strength from mid-zygotene to early 1-pachytene may reflect the progression of the DSB repair events with the depletion of H3K4me3 once the SEI starts to form.

Interestingly, our analysis of H3K4me1 revealed a similar trend but different dynamics. We found that very weak but detectable H3K4me1 signals started to appear even in type B spermatogonia; however, substantial H3K4me1 signals occurred in mid-preleptotene, and persisted until mid-pachytene, which lasted significantly longer than H3K4me3 signals. Although H3K4me1 peaks in mid-zygotene, similar to H3K4me3, we found that it covered a longer range, likely more nucleosomes, than H3K4me3 did (Fig. 2c; Supplementary information, Fig. S8f). Interestingly, most central DSB nucleosomes were converted from H3K4me1 to H3K4me3 in mid-zygotene and lasted till late-zygotene (Fig. 2c), consistent with the H3K4me3 pattern described above. Such patterns indicated that the chromatin containing DSB is subjected to a previously unappreciated dynamic regulation between H3K4me1 and H3K4me3, which might also be mediated by PRDM9. Supporting this, our in vitro enzymatic analyses found that PRDM9 mainly catalyzed generation of H3K4me1 and H3K4me3 in recombinant nucleosomes (Fig. 2d). Such intrinsic enzymatic features indicate that PRDM9-mediated H3K4me1 may also functionally contribute to DSB formation.

Altogether, these results reveal a specific spatiotemporal regulation of the hotspot-associated H3K4me3 dynamics during meiosis prophase I, which is intrinsically coupled with a previously unknown transition from H3K4me1 to H3K4me3 mediated by PRDM9, and indicate that they may play a role in the progression of recombination.

### PRDM9-mediated H3K4me3 signals facilitate the fate of DSBs

**Fig. 3:**
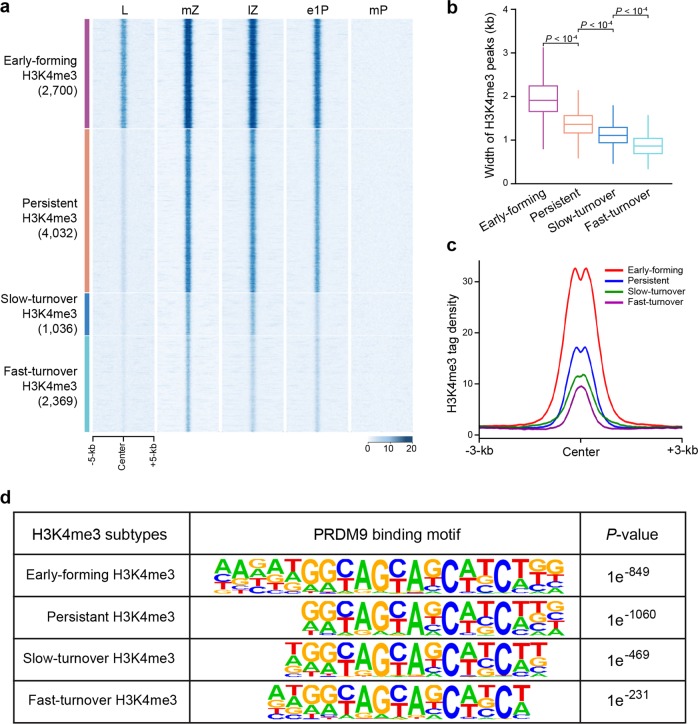
The properties of hotspot-associated H3K4me3 marks during meiotic recombination. **a** Heatmaps of H3K4me3 normalized tag density on four types of hotspot-associated H3K4me3 peaks with distinct turnover times from leptotene to middle pachytene spermatocytes. Each row represents a DSB hotspot region of ±5 kb around the center. L leptotene, mZ mid-zygotene, lZ late-zygotene, e1P early 1-pachytene, mP mid-pachytene. **b** Boxplot showing the average width of the four subtypes of hotspot-associated H3K4me3 peaks in mid-zygotene. Definitions of the four subtypes of hotspot-associated H3K4me3 are detailed in Materials and Methods. **c** Profile of the averaged H3K4me3 tag density at mZ stage on the four subtypes of hotspot-associated H3K4me3 peaks. H3K4me3 tag density was calculated using H3K4me3 read coverage with 50-bp resolution. mZ mid-zygotene spermatocytes. **d** Identified PRDM9 motifs enriched on four subtypes of hotspot-associated H3K4me3 peaks.

Little is known about the potential roles of PRDM9-mediated H3K4me3 during DSB repair; particularly how and when the cell determines which DSBs will be repaired as COs or NCOs remains unclear. Notably, we found that of the 10,137 hotspot-associated H3K4me3 marks generated specifically in mid-zygotene, 2369 were quickly erased during the mid-late zygotene stage (henceforth defined as fast-turnover H3K4me3), and a further 1036 were erased during the late-zygotene to early 1-pachytene stage (defined as slow-turnover H3K4me3); this period, from the mid-zygotene to early 1-pachytene stage, corresponds to DSB disappearance and NCO formation (Fig. 3a)^9,10,34,35^. Moreover, only 3 of the 7375 hotspot-associated H3K4me3 peaks persisting in early 1-pachytene were observed in mid-pachytene, during which the SEIs are thought to be converted to dHJs^9,10,34,35^. We therefore inferred that DSBs with H3K4me3 marks that disappear more quickly tend to be repaired as NCOs by the SDSA pathway during the zygotene stage, whereas more persistent H3K4me3 marks more likely denote sites of CO-designated recombination during the early-mid pachytene stage. These results indicated that the dynamics of the hotspot-associated H3K4me3 is in accordance with the reported kinetics of DSB formation and repair, such as the onset of SEI formation and the onset of dHJ formation^34–36^. It is tempting to speculate that hotspot-associated H3K4me3 generated at different times are distinct from one another, and that these varying types create corresponding environments that support either NCO differentiation or CO formation.

To test this hypothesis, we grouped the hotspot-associated H3K4me3 marks of mid-zygotene into four types according to their half-life: early-forming H3K4me3, fast-turnover H3K4me3, slow-turnover H3K4me3, and persistent H3K4me3 (persisting in early 1-pachytene but excluding early-forming H3K4me3; Supplementary information, Table S5). A striking feature was that the average width around the hotspot midpoint, which may reflect the breadth of DSB resection engaged in recombination, was the widest for early-forming H3K4me3 (~2 kb), and the narrowest for fast-turnover H3K4me3 peaks (<1 kb) (Fig. 3b). This is consistent with the data showing that hotspots with greater H3K4me3 signals have more COs, and hotspot-associated H3K4me3 signals could limit dHJ migration^27,35,37,38^. Furthermore, the average strength of the H3K4me3 signals was again the highest for early-forming H3K4me3, and the lowest for fast-turnover H3K4me3 (Fig. 3c). In addition, by motif analyses, we found that although the Prdm9 binding motif was significantly enriched in all four groups, the early-forming group was enriched for a significantly longer Prdm9 motif (with extended 5′ and 3′ consensus). This indicates that the underlying DNA sequence plays a role in determining preferential Prdm9 binding at the early-forming DSB sites consisting of the stronger motifs (Fig. 3d). Taken together, these lines of evidence indicate that stronger, more extended, and longer-lasting hotspot-associated H3K4me3, which are features associated with early-forming marks, may create a more stable and permissive chromatin environment, possibly by favoring the recruitment of the CO designation proteins such as pro-crossover proteins to prompt the dHJ pathway, thereby directing DSBs toward a CO outcome. Alternatively, short-lasting and weaker H3K4me3 signals likely shepherd DSBs toward an NCO fate. Previous studies had suggested that the control of CO/NCO differentiation occurs during or prior to zygotene^9,34,36,39,40^. We provide evidence that CO/NCO differentiation might take place at the time of DSB formation as early as leptotene, and that PRDM9-mediated H3K4me3 (the newly generated H3K4me3 at leptotene and mid-zygotene stages) could facilitate DSB fate by coupling DSB formation with the CO/NCO differentiation control. Indeed, it has recently been suggested that PRDM9 may influence the probability that a DSB will be repaired by a CO^38^. Accordingly, the hotspot-associated H3K4me3 marks that overlapped with 14 of the 16 well-known CO hotspots were already present in leptotene and persisted through zygotene and early 1-pachytene (Supplementary information, Fig. S9)^26,41,42^.

### Several epigenetic factors influence meiotic recombination

**Fig. 4:**
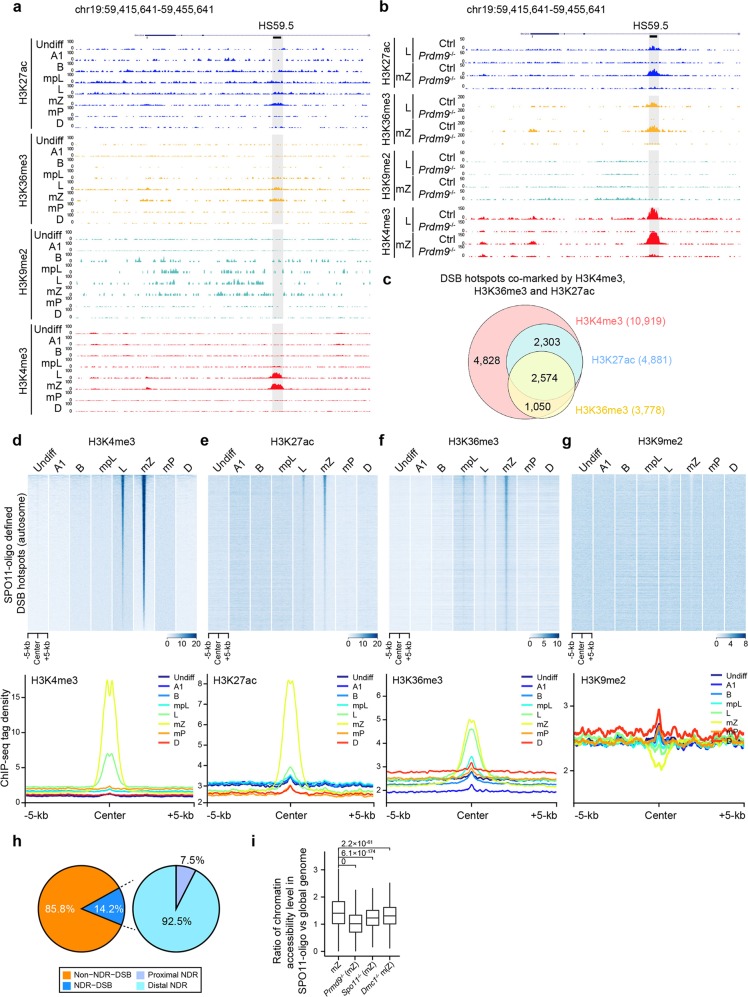
Specific epigenetic marks associated with recombination hotspot formation. **a** UCSC genome browser view of the ChIP-seq read coverage at a known CO hotspot; H3K27ac, H3K36me3, H3K9me2, and H3K4me3 (as control) ChIP-seq data were included. The known DSB hotspot and CO hotspot (HS59.5) were highlighted with light black shading. Region shown represents chromosome 19 (chr19): 59,415,641–59,455,641. **b** UCSC genome browser view of the ChIP-seq read coverage at the same known CO hotspot as **a** in leptotene and mid-zygotene spermatocytes of *Prdm9*^−/−^ mice; H3K27ac, H3K36me3, H3K9me2, and H3K4me3 (as control) ChIP-seq data were included. **c** Venn diagram showing the overlap of hotspot-associated H3K4me3, H3K36me3, and H3K27ac peaks in mid-zygotene spermatocytes. **d**–**g** Heatmaps (top) and profiles (bottom) of H3K4me3 (**d**), H3K27ac (**e**), H3K36me3 (**f**), and H3K9me2 (**g**) on the SPO11-oligo defined DSB hotspots in spermatogenic cells. Each row in heatmaps represents a DSB hotspot of ±5 kb around the center and ranked by H3K4me3 tag density from the highest to the lowest. Color indicates ChIP-seq tag density. Average ChIP-seq tag density was calculated using ChIP-seq reads with 50-bp resolution. **h** Pie chart showing that the minority of DSB hotspots are positioned within NDRs, most of which are distal NDRs. **i** Boxplot showing the ratio of chromatin accessibility level (chromatin accessibility level in SPO11-oligo defined DSB hotspots versus that in the whole genome) in the WT, *Prdm9*^−/−^, *Spo11*^−/−^, and *Dmc1*^−/−^ mid-zygotene spermatocytes. Only regions with at least three GCH sites were considered. Undiff undifferentiated spermatogonia, A1 type A1 spermatogonia, B type B spermatogonia, mpL mid-preleptotene spermatocytes, L leptotene spermatocytes, mZ mid-zygotene spermatocytes, mP mid-pachytene spermatocytes, D diplotene spermatocytes, Ctrl control.

We analyzed whether other chromatin features could affect meiotic recombination. Both H3K36me3, another catalytic product of PRDM9^43–45^, and H3K27ac, also showed similar, albeit weaker, overlapping patterns with DSB hotspots to H3K4me3 (Fig. 4a). In contrast, the H3K9me2 signal showed a moderate but significant depletion at DSB hotspots (Fig. 4a). However, there was no discernible correlation between DSB hotspots and H3K27me3, or H3K9me3 (Supplementary information, Fig. S10a, b). The H3K36me3 and H3K27ac marks overlapping with DSB hotspots were all newly generated in leptotene and zygotene, similar to H3K4me3. We observed that 2574 DSB hotspots are co-marked by H3K4me3, H3K36me3, and H3K27ac modifications (Fig. 4c–f). In contrast, the H3K9me2 peaks were specifically depleted around hotspots in leptotene and zygotene, but present before the meiotic stages (i.e., in spermatogonia and preleptotene spermatocytes) (Fig. 4g). Most of the newly generated H3K27ac and H3K36me3 peaks and the depleted H3K9me2 regions overlapped with the newly generated H3K4me3 peaks, suggesting that they may be co-regulated and function together to orchestrate DSB hotspots (Supplementary information, Table S6). The average strengths of the newly generated H3K27ac and H3K36me3 marks were also weaker than those of the common ones, similar to the newly generated H3K4me3 marks (Supplementary information, Fig. S11a, b). Again, most of the newly generated H3K27ac and H3K36me3 signals in the WT were absent in *Prdm9*^−/−^ spermatocytes, and the focal H3K9me2 depletion was also blocked by loss of *Prdm9* (Fig. 4b). Furthermore, the levels and distribution of H3K27ac, H3K36me3 and H3K9me2 were similar in the WT, *Spo11*^−/−^ and *Dmc1*^−/−^ mice, consistent with those of the newly generated H3K4me3 (Supplementary information, Fig. S12a–c). Altogether, these results reveal that the addition of H3K27ac and H3K36me3, and the focal loss of H3K9me2, are all specifically coupled to *Prdm9*, and are likely involved in determining a subset of DSB hotspots together with H3K4me3. It remains to be determined how *Prdm9* affects these histone modifications, especially H3K27ac and H3K9me2.

Previous studies from different labs have generated conflicting results regarding the association of DSB hotspot centers with nucleosome occupancy^20,26,27^. We found that most recombination hotspots (85.8%) were positioned on chromatin packaged into nucleosomes, whereas only a minority (14.2%) of the DSB hotspots originated from loci within nucleosome-depleted regions (NDRs), with the majority being distal NDRs, i.e., >1.5 kb from a transcription start site (TSS) (Fig. 4h; Supplementary information, Table S7). Thus, our data suggest that DSBs are not constrained by NDRs in mice, as they are in fission yeast^46^. Although most recombination hotspots were positioned with nucleosomes, the chromatin accessibility around them in zygotene was much more open compared with that in global genomic regions (Supplementary information, Fig. S13a). The hotspots within NDRs were much stronger than those outside NDRs (Supplementary information, Fig. S13b), while the H3K4me3 signals around NDR-DSBs were also stronger than those around non-NDR-DSBs (Supplementary information, Fig. S13c). Notably, in the *Prdm9*^−/−^, *Spo11*^−/−^, or *Dmc1*^−/−^ zygotene, the chromatin accessibility in the DSB hotspots (defined by SPO11-oligo) was significantly weaker than that in WT zygotene (Fig. 4i; Supplementary information, S13d). However, in the *Prdm9*^−/−^ zygotone, the chromatin accessibility in the DSB hotspots (defined by ssDNA in *Prdm9*^−/−^ spermatocytes) was significantly stronger than that in WT, *Spo11*^−/−^, or *Dmc1*^−/−^ zygotene^47^ (Supplementary information, Fig. S13e, f). These results indicated that the chromatin accessibility level in hotspots, unlike the histone marks, is dependent on all three factors, *Prdm9*, *Spo11*, and *Dmc1*, and, therefore, could be a consequence of DSB formation and/or repair. However, we cannot exclude the possibility that spermatocytes were arrested since the *Spo11* and *Dmc1* mutations might affect global chromatin composition and organization.

We also analyzed the DNA methylation levels in the DSB hotspots (defined by SPO11-oligo). We found that the DNA methylation levels around the DSB hotspots are similar to those in global genomic regions, suggesting that DNA methylation may not be involved in DSB formation (Supplementary information, Fig. S13g).

### Open chromatin states contribute to the fate decision of DSBs

**Fig. 5:**
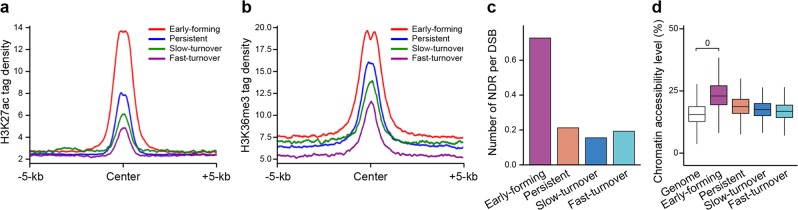
Chromatin states around the four subtypes of hotspot-associated H3K4me3 peaks. **a, b** Profiles of the averaged H3K27ac (**a**) and H3K36me3 (**b**) tag density on the four subtypes of hotspot-associated H3K4me3 peaks at mid-zygotene. H3K27ac and H3K36me3 tag densities were calculated using ChIP-seq read coverage with 50-bp resolution. **c** The average number of NDRs per DSB ±2 kb at the sites of four subtypes of hotspot-associated H3K4me3 peaks. NDRs were defined in mid-zygotene spermatocytes. **d** Boxplots showing the chromatin accessibility level in five regions, early-forming H3K4me3 regions, persistent H3K4me3 regions, slow-turnover H3K4me3 regions, fast-turnover H3K4me3 regions, and whole-genome regions excluding these four cluster regions. Only regions with at least three GCH sites were considered.

We further tested whether H3K27ac, H3K36me3, and chromatin state contributed to recombinational progression. We found that the width and density of H3K27ac and H3K36me3 peaks around the four groups of DSBs described above (Fig. 3c) were similar to those of the H3K4me3 peaks (Fig. 5a, b), indicating that the DSBs at sites of early-forming H3K4me3 also occupy extended and stronger H3K27ac and H3K36me3 signals. Moreover, DSB regions with early-forming H3K4me3 were also significantly enriched for NDRs (Fig. 5c), further confirming a connection between the active histone modification and open chromatin state at DSB sites. We then analyze the chromatin accessibility around PRDM9-binding sites (defined by the PRDM9-affinity-Seq) in these four clusters^29^. The results showed that there is a significant difference for chromatin accessibility between global genomic regions and early-forming H3K4me3 regions in zygotene stages. For the other three clusters, the differences are milder (Fig. 5d). In addition, the chromatin state around the 16 well-known CO hotspots (described above) was much more open than that around general DSB hotspots (defined by SPO11-oligo), or around the DSBs at the sites of fast-turnover, slow-turnover, or even persistent H3K4me3 marks (Supplementary information, Fig. S14). Taken together, these results indicate that H3K27ac and H3K36me3, coordinately with PRDM9-mediated H3K4me3, generate a robust and permissive chromatin environment at DSBs, in particular, the earlier formed DSBs, to support them to proceed to a CO event.

## Discussion

**Fig. 6:**
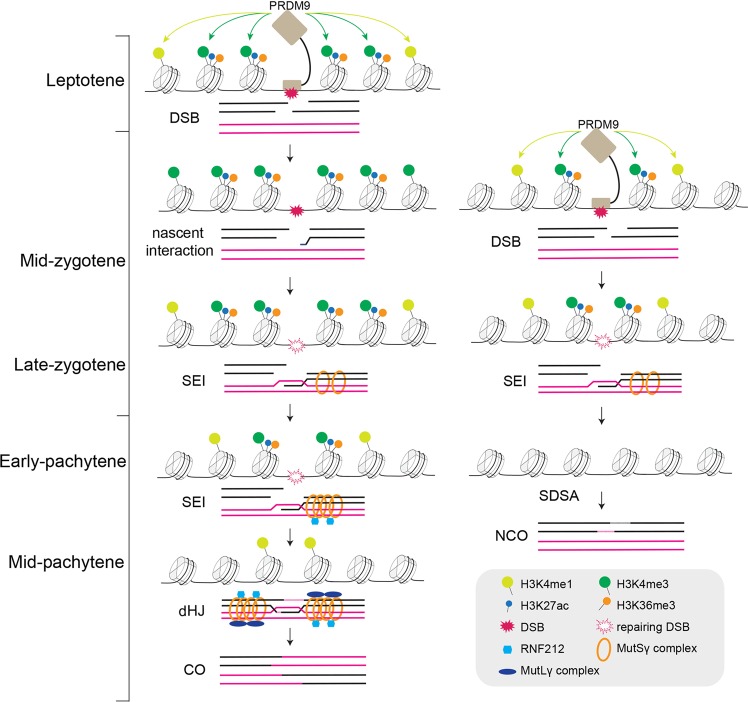
Proposed model for the DSB fate decision. At an earlier stage, e.g., leptotene, PRDM9 binds to its target sites containing a longer and stronger binding motif, where it catalyzes the deposition of stronger and extended H3K4me3, and probably directs the deposition of H3K36me3 and H3K27ac. These stronger and more extended H3K4me3 marks may create a stable and permissive chromatin “niche” to predesignate DSB sites to follow a CO fate that facilitates the recruitment of pro-crossover proteins. In contrast, at a later stage, PRDM9 drives deposition of weaker and narrower H3K4me3 marks around its binding sites. The DSBs at the sites of these weaker and narrower H3K4me3 modifications are more likely repaired as NCOs.

Here we characterize the dynamics of PRDM9-mediated H3K4me3 and other epigenetic factors during meiotic prophase I, and suggest that the majority of mouse DSBs might occur during zygotene. We propose that the DSB fate decision is made at an early step in recombination (Fig. 6). Given the complication of controlling the DSB fate, the exact role of PRDM9-mediated H3K4me3 on CO/NCO differentiation will require further direct examination of the link between faster turnover hotspot-associated H3K4me3 and NCO events or between persistent hotspot-associated H3K4me3 and CO events. CO homeostasis maintains CO numbers with variation in DSB numbers in yeast, worms, mice, and probably humans, at the expense of NCOs^33,48–51^. We note several implications for this model in the control of CO homeostasis. First, the earliest formed DSBs in leptotene occupy stronger, extended, and long-lasting hotspot-associated H3K4me3, and more likely proceed toward CO, providing evidence that exquisite homeostatic control of CO occurs at the beginning point of meiotic recombination. In principle this control could ensure sufficient CO-designated DSB numbers even if dysfunction of the DSB-forming machinery, e.g., by the *Spo11* hypomorphic mutation, results in fewer DSBs^33^. Second, we and others have shown that a subset of PRDM9-mediated H3K4me3 modifications do not drive DSB generation, indicating that PRDM9 activity usually exceeds the amount required for DSB formation^43^. However, PRDM9-mediated H3K4me3 that do not overlap with DSB hotspots displayed weak and short peaks similar to the fast-turnover H3K4me3 (Supplementary information, Figs. S7, S15a, b). Even if too many DSBs were generated at the sites of these excessive PRDM9-mediated H3K4me3 modifications, e.g., as in *Spo11* transgenic mice and ATM^−/−^ mice, they will be more likely to be repaired as NCOs^33,52^. Thus, excessive PRDM9-mediated H3K4me3 modifications could provide “buffering” capacity to guarantee CO homeostasis. Third, our findings could serve as basis for future studies on how pro-crossover factors localize to specific recombination sites by recognizing epigenetic environment^39^. The PRDM9-mediated H3K4me3 marks along with other epigenetic modifications (i.e., H3K27ac and H3K36me3) coordinately generate a robust and permissive chromatin ‘niche’ to predesignate DSB sites as CO sites that license the local recruitment of pro-crossover factors (Fig. 6). It will be of interest to test this proposed model in the future.

Our findings highlight potentially crucial roles of PRDM9-mediated H3K4me3 in directing DSB fate, provide new insight into CO homeostatic control, and call for future studies on how PRDM9 selects chromatin regions at an early stage to generate stronger and extended H3K4me3.

## Materials and methods

### Mice

Mice used in this study were as follows: *Lin28-*YFP, *Vasa-*mCherry, *Stra8-*GFPCre, *Prdm9*-3×(HA-Flag), *Prdm9*^−/−^ (*Prdm9*^tm1Ymat^), *Spo11*^−/−^ (*Spo11*^tm1Mjn^), and *Dmc1*^−/−^ (*Dmc1*^tm1Jcs^). The *Lin28-*YFP, *Vasa-*mCherry and *Stra8-*GFPCre mice were described previously^25^. The *Prdm9*-3×(HA-Flag) knockin mouse line was generated by the CRISPR/Cas9 technology. A cDNA encoding the 3×(HA-Flag) tag was inserted into the N-terminus of *Prdm9*. The *Prdm9*-3×(HA-Flag) line was generated by Shanghai Biomodel Organism Co., Ltd. The *Lin28-*YFP, *Vasa-*mCherry, *Stra8-*GFPCre, and *Prdm9*-3×(HA-Flag) lines were all maintained on the C57BL/6J (B6) background. The *Prdm9*^tm1Ymat^ mice were obtained from RIKEN BioResource Center. The *Spo11*^tm1Mjn^ and *Dmc1*^tm1Jcs^ mice were obtained from the Jackson Laboratory (Stock#: 019117 and 008608). The *Prdm9*^tm1Ymat^, *Spo11*^tm1Mjn^, and *Dmc1*^tm1Jcs^ mice were backcrossed into the C57BL/6J (B6) for five generations. All animal experiments were conducted in accordance with the guidelines in the Animal Care and Use Committee at Center for Excellence in Molecular Cell Science (CEMCS), Shanghai Institute of Biochemistry and Cell Biology (SIBCB), Chinese Academy of Sciences.

### Spermatogenesis synchronization

Spermatogenesis was synchronized and validated as previously described^25^. Briefly, 2-dpp mice were pipette fed 100 μg/g body weight WIN 18,446 (MP), suspended in 1% gum tragacanth, for seven consecutive days. On Day 8 of WIN 18,446 treatment, these animals were received an i.p. injection of retinoic acid (Sigma; 25 μg/g body weight) in dimethyl sulfoxide (DMSO), and were then left to recover for sample collections.

### Isolation of spermatogenic cells

The undifferentiated spermatogonia were isolated from *Lin28-*YFP knockin mice. Testes from 3-week-old *Lin28-*YFP knockin mice were collected and digested by type I collagenase and 0.25% Trypsin as described previously^25^. After centrifugation, the pellet was resuspended in DMEM containing 2% BSA at a concentration of 1 × 10^6^ cells/40 μL, followed by incubation with PE-conjugated anti-CD117 antibody (0.1 μg/10^6^ cells; Molecular probes) for 30 min on ice. The undifferentiated spermatogonia (YFP-positive, PE-negative population) were collected using FACS (BD). The type A1 spermatogonia were isolated from synchronous *Stra8-*GFPCre mice. Testes were collected and digested. The type A1 spermatogonia (GFP-positive population) were collected at given time-point 10 h after retinoic acid treatment using FACS. The type B spermatogonia and spermatocytes at different stages were isolated from synchronous *Lin28-*YFP and *Vasa-*mCherry double-positive mice. To isolate the type B spermatogonia, testes were digested and the synchronous advanced spermatogenic cells (mCherry-positive population) were collected at given time-point 135 h after retinoic acid treatment using FACS. To isolate spermatocytes at different stages, testes were digested and the cell suspensions were stained with Hoechst 33342. The mCherry-positive 2N–4N or 4N populations were collected using FACS. Specifically, spermatocytes at different stages were collected at given time-points 168 h (mid-preleptotene, mpL), 186 h (leptotene, L), 196 h (early-zygotene, eZ), 208 h (mid-zygotene, mZ), 216 h (late-zygotene, lZ), 240 h (early 1-pachytene, e1P), 276 h (early 2-pachytene, e2P), 312 h (mid-pachytene, mP), and 384 h (diplotene, D) after retinoic acid treatment. The identities of sorted spermatocytes were validated using nuclear spreading and immunofluorescence.

### Nuclear spreading and immunofluorescence

Nuclear spreading and immunofluorescence were performed as described^53^. For immunofluorescence analysis, the following primary antibodies were used: rabbit anti-SYCP3 (Abcam), mouse anti-γH2AX (Millipore), rabbit anti-HA (Cell Signaling Technology (CST)), and mouse anti-SYCP3 (Santa Cruz). The spreading nuclei were then detected with Alexa Fluor 488-conjugated or 594-conjugated secondary antibodies (Jackson ImmunoResearch), mounted, and analyzed by fluorescence microscopy.

### NOMe-seq library preparation and sequencing

NOMe-seq assay was performed as previously described with some modifications^54^. Briefly, ~1000 cells were transferred into a 0.2 μL PCR tube containing 7 μL of ice-cold lysate buffer (50 mM Tris-HCl, pH 7.4, 50 mM NaCl, 0.25 mM EDTA, 10 mM DTT, 0.25 mM PMSF and 0.5% NP-40, plus 2 pg λDNA). After gently vortexing, the cell lysate was kept on ice for 10 min. The GpC methyltransferase M. CviPI and SAM were then added to the lysate to a final volume of 10 μL containing 1 U/μL M. CviPI and 160 μM SAM. The in vitro methylation of nuclei was performed by incubating the mixture in a thermocycler at 37 °C for 45 min followed by heating at 65 °C for 25 min to inactive the enzyme activity. After in vitro methylation, 1 μL of 20 mg/mL protease (Qiagen) was added and the mixture was incubated for 3 h at 50 °C to release genomic DNA. The released genomic DNA was then bisulfite converted using the EZ-96 DNA Methylation-Direct MagPrep (Zymo) according to the manufacturer’s instructions. Afterward, the purified DNAs were annealed using random nonamer primers with a 5′-biotin tag (5′-Biotin-CTACACGACGCTCTTCCGATCTNNNNNNNNN-3′) in the presence of Klenow fragments (3′-5′ exo-, NEB). Then, the primers were digested by exonuclease I (NEB) and the DNA was purified using Agencourt Ampure XP beads (Beckman Coulter). Dynabeads M-280 Streptavidin (Invitrogen) were then used to immobilize the newly synthesized biotin-tagged DNA strands, and the original bisulfite-converted DNA templates were removed. Second DNA strands were synthesized using Klenow fragment with random nonamer primers (5′-AGACGTGTGCTCTTCCGATCTNNNNNNNNN-3′). After washing, the beads were used to amplify libraries using 15 cycles of PCR with the universal primer and index primer (NEB). The amplified libraries were purified with Agencourt Ampure XP beads twice. Fragments from 300 to 800 bp were selected by argarose gel electrophoresis and purified by Zymoclean Gel DNA Recovery Kit (Zymo). Finally, libraries were pooled and sequenced on the Illumina HiSeq 2500 sequencer for 150-bp paired-end sequencing.

### ChIP

ChIP assay was carried out as previously described^55^. The sorted homogenous synchronous spermatogenic cells were crosslinked with 1% formaldehyde for 10 min and then stopped by adding 125 mM glycine. Chromatin samples were lysed with lysis buffer (20 mM Tris-HCl, pH 8.0, 500 mM NaCl, 1 mM EDTA, 1% Triton X-100, and 0.1% SDS) and sonicated with Qsonica. Histone modification specific antibody was incubated with chromatin samples overnight at 4 °C. Antibodies used were as follows: H3K4me3 (CST #9751s, Lot: 10), H3K9me2 (Abcam #Ab1220, Lot: GR325223-4), H3K9me3 (Active motif #39161, Lot: 15617003), H3K27ac (Active motif #39133, Lot: 20017009), H3K27me3 (CST #9733s, Lot: 8), H3K36me3 (CST #4909s, Lot: 2). 5 × 10^4^ cells were used for H3K4me3 and H3K27ac ChIP. 1 × 10^5^ cells were used for H3K27me3 and H3K36me3 ChIP. 2 × 10^5^ cells were used for H3K9me2 and H3K9me3 ChIP. 0.5 μg spike-in antibody (Active motif #61686) and 25 ng spike-in chromatin (Active motif #53083) were used in this ChIP assay according to the manufacturer’s guidelines. The protein-DNA complexes were immobilized on 15 μL protein A/G beads (Smart lifesciences SA032005) and then washed four times with lysis buffer, twice with low salt buffer (10 mM Tris-HCl, 250 mM LiCl, 1 mM EDTA, 0.5% NP-40, 0.5% Na-deoxylcholate) and once with 10 mM Tris-HCl, pH 8.0. Decrosslinking was carried out in elution buffer (50 mM Tris-HCl, pH 8.0, 10 mM EDTA and 1% SDS) at 65 °C for 5 h. Proteinase K and RNase A digestions were performed at 55 °C for 1 h. DNA samples were purified with PCR extraction kit (QIAGEN #28006). DNA samples were analyzed using real-time PCR and prepared for deep sequencing according to the manufacturer’s guidelines (KAPA Biosystems KK8503 and VAHTS Universal DNA Library Prep Kit for Illumina V3 ND607). Finally, libraries were pooled and sequenced on the Illumina HiSeq 2500 sequencer for 150-bp paired-end sequencing.

### Histone methyltransferase assay and mass spectrometry

The histone methyltransferase assay was carried out overnight in 20 μL system containing 50 mM Tris-HCl, pH 8.5, 30 mM NaCl, 50 μM SAM, 1 mM TCEP, 5% glycerol, 400 ng of GST-tagged full-length mPrdm9 bound on 5 μL GSH beads, and 1 μg of H3.1 oligonucleosomes, at room temperature. After the reaction, half of the system was separated by 12.5% SDS-PAGE and stained with CBB. The SDS-PAGE gel containing histone H3.1 was cut out, destained, added with propionyl in 25 mM Ammonium bicarbonate (ABC), 25% ACN, 100 mM NHS at 50 °C for 30 min, and then digested in-gel at 37 °C for 16 h, with 10 ng/μL trypsin in 25 mM ABC. The peptides were extracted by extraction buffer (5% formaldehyde, 50% ACN), subjected to propionylation again, and then examined by MRM mass spectrometry. Each sample was repeated for three times.

### Processing NOMe-seq data

Raw NOMe-seq sequencing reads were firstly subjected to trimming of 9-bp random primer, and removal of adapters and low-quality bases using *trim_galore* (version: 0.1.3) with parameters ‘*–quality 20 –stringency 3 –length 50 –clip_R1 9 –clip_R2 9 –paired –trim1 –phred33*’. Clean reads were mapped against mouse reference genome mm10 (UCSC) using *Bismark* (version: 0.7.6) with a paired-end and non-directional mode (parameters ‘*–fastq –non_directional –unmapped –phred33-quals*’)^56^. To improve the number of mapped reads, the unmapped reads were realigned to the same reference genome in a single-end aligned mode. After alignment, final BAM files were obtained when PCR duplicated reads were removed using *SAMtools* (version: 0.1.18)^57^.

After detecting the biological replication, we merged final BAM files of the same replication. We used 3× as the read depth cut-off in the downstream analysis. We defined the methylation level of each detected cytosine site as the number of methylated reads ‘C’ divided by the number of all detected reads (methylated and unmethylated reads, ‘C+T’). We used WCG (ACG/TCG) for DNA methylation analysis and GCH (GCA/GCC/GCT) for chromatin accessibility analysis. In this study, only regions with at least three WCG/GCH sites were considered, which was indicated in each figure legend.

### Identification of NDRs

NDRs were regions with significantly higher GCH levels than that in the whole genome background. We identified NDRs with 3× coverage GCH sites for each sample. We detected the average GCH methylation level in a 100-bp window with a 20-bp sliding step, and only regions matched the following three measures were defined as NDRs: (1) the average GCH methylation level significantly higher than the whole genome background with *P* ≤ 10^−10^ by χ^2^ test; (2) the number of GCH sites > 5; and (3) the length > 140 bp.

We defined NDRs as proximal NDRs and distal NDRs. NDRs located within 1.5 kb upstream and 1.5 kb downstream of the TSS were considered as proximal NDRs, and otherwise, as distal NDRs.

### ChIP-seq data processing

Firstly, all of the histone modification ChIP-seq data were sent to *trim_galore* to remove adapter contamination and low-quality sequences^58^; Phred score of any bases in a read under 20 and adapter sequence were removed, and reads after trimming shorter than 30 were discarded. Trimmed sequences were then mapped with *Bowtie2* (version: 2.2.6) using default parameters^59^. Duplicate reads were removed by *SAMtools* (‘*samtoolsrmdup -s*’). Unique and monoclonal mapped reads were extended to 150 bp based on the average sonicated chromatin DNA length. The spike-in reads were mapped to Drosophila genome (dm6) using the same parameters. H3K4me3 and H3K27ac peaks were called using *MACS14* with default parameters (*P* value = 1e^−5^)^60^; H3K27me3, H3K36me3, and H3K9me3 peaks were called using *MACS2* with broad peak mode and default settings (q value = 0.01). *genomeCoverageBed* (*bedtools*) was used to transfer BED files to bedGraph files^61^, and then spike-in read was used as a normalization factor that equalizes the signal across samples. Normalized bedGraph files were subsequently transferred to bigwig files which were used to generate the heatmap and metagene profile on regions of interest. We used *deepTools* to perform the histone modification distribution analysis (heatmap and profile)^62^.

### Heatmap and profile for ChIP-seq

The heatmap for histone modification scores associated with genomic regions on regions of interest (DSB hotspots) was generated by *computeMatrix* and *plotHeatmap* modules in *deepTools*. Firstly, we used *computeMatrix* to generate a matrix file with a normalized bigwig file on DSB hotspots, and used *plotHeatmap* to create the heatmap in which DSB hotspots were sorted by SPO11-oligo density from the highest to the lowest.

The profile for histone modification scores associated with genomic regions on regions of interest (DSB hotspots) was generated by *computeMatrix* and *plotProfile* modules in *deepTools*. Matrix files were generated using the same method as the heatmap, and then the profile was created by *plotProfile*.

### De novo and common H3K4me3 peak identification

De novo H3K4me3 peaks were identified by filtering the H3K4me3 peaks in leptotene and mid-zygotene spermatocytes which are not present at previous stages of homogeneous spermatogenic cells. The other H3K4me3 peaks in leptotene and mid-zygotene spermatocytes were defined as common H3K4me3 peaks. De novo H3K27ac and H3K36me3 peaks were identified using the same method as de novo H3K4me3 peaks.

### Identification of H3K9me2 dip regions

H3K9me2 dip regions were identified on DSB hotspots. We used twice length of DSB hotspots to extend regions in both sides of DSBs (left and right regions around DSBs), and compared the average H3K9me2 density in DSB hotspots and their both sides. Only DSB hotspots with average H3K9me2 density less than twice of their both sides were defined as H3K9me2 dip regions.

### Definition of DSB hotspots

DSB hotspots used in this study were defined by SPO11-oligo maps from WT C57BL/6J mice, which were collected from the GEO database under the accession number: GSE84689. DSB hotspots are defined as SPO11-oligo clusters using cutoffs described in the previous study^26^. DSB hotspots in *Prdm9*^−/−^ C57BL/6J mice were defined by anti-DMC1 ssDNA sequencing data^20^, which were collected from GEO under the accession number: GSE35498. Only DSB hotspots in autosomes were used for downstream analysis.

### Collection of PRDM9-affinity-seq data

PRDM9-affinity-seq data in mouse spermatocytes were collected from the GEO database under the accession number: GSE61613.

### Motif analysis for de novo and common H3K4me3 peaks

We searched for enrichment of DNA sequence motifs within de novo and common H3K4me3 peaks using the motif discovery module in *homer* software (parameters ‘*findMotifsGenome.pl -size 750 -len 8,10,15,20 -mask -cpg’*)^63^. Only top 3000 de novo and common H3K4me3 peaks were considered for motif analysis based on *P* values of H3K4me3 peaks.

### Classification of DSB hotspot-associated H3K4me3 peaks

We classified DSB hotspot-associated H3K4me3 peaks into four clusters based on the time of appearance and disappearance of H3K4me3 peaks on DSB hotspots. First, 2700 hotspot-associated H3K4me3 peaks that start to appear in leptotene stage were defined as early-forming H3K4me3. Second, we compared hotspot-associated H3K4me3 peaks in leptotene, mid-zygotene, and late-zygotene, and 2369 hotspot-associated H3K4me3 peaks existing in mid-zygotene but not leptotene and quickly erased in late-zygotene were defined as fast-turnover H3K4me3. Third, we compared hotspot-associated H3K4me3 peaks in leptotene, mid-zygotene, late-zygotene and early-pachytene, 1036 hotspot-associated H3K4me3 peaks existing in mid-zygotene and late-zygotene but not leptotene and then quickly erased in early-pachytene were defined as slow-turnover H3K4me3. Finally, the other 4032 hotspot-associated H3K4me3 peaks existing in mid-zygotene but not leptotene and then retained in late-zygotene and early-pachytene were defined as persistent H3K4me3.

### Calculation of the width of H3K4me3 peaks

The width of H3K4me3 peaks was calculated by subtracting start coordinates from end coordinates of the H3K4me3 peak output by peak caller.

### Quantification and statistical analyses

Statistical analyses were performed using R versions (http://www.r-project.org). Fisher’s exact test was used to test whether two sets of peaks are related spatially. Unpaired *t*-test was used to compare the peak length of the four subtypes of DSB hotspot-associated H3K4me3 peaks. Two-tailed Student’s *t*-test was used to compare the difference of chromatin accessibility levels.

## Supplementary information

Supplementary information, Figure S1

Supplementary information, Figure S2

Supplementary information, Figure S3

Supplementary information, Figure S4

Supplementary information, Figure S5

Supplementary information, Figure S6

Supplementary information, Figure S7

Supplementary information, Figure S8

Supplementary information, Figure S9

Supplementary information, Figure S10

Supplementary information, Figure S11

Supplementary information, Figure S12

Supplementary information, Figure S13

Supplementary information, Figure S14

Supplementary information, Figure S15

Supplementary information, Table S1

Supplementary information, Table S2

Supplementary information, Table S3

Supplementary information, Table S4

Supplementary information, Table S5

Supplementary information, Table S6

Supplementary information, Table S7

## Data Availability

The raw data and processed data have been deposited in the Gene Expression Omnibus (GEO, https://www.ncbi.nlm.nih.gov/geo/) under the accession number: GSE132446.

## Code availability

Source codes are available upon request.
