# Supplementary material for: Refined spatial temporal epigenomic profiling reveals intrinsic connection between PRDM9-mediated H3K4me3 and the fate of double-stranded breaks
Source: Cell Res. 2020 Feb 11;30(3):256–68. doi: 10.1038/s41422-020-0281-1 (PMC7054334; doi:10.1038/s41422-020-0281-1)
Supplement: Supplementary file 1 — Supplementary information, Figure S1 [file 41422_2020_281_MOESM1_ESM.pdf]

Supplementary information, Figure S1

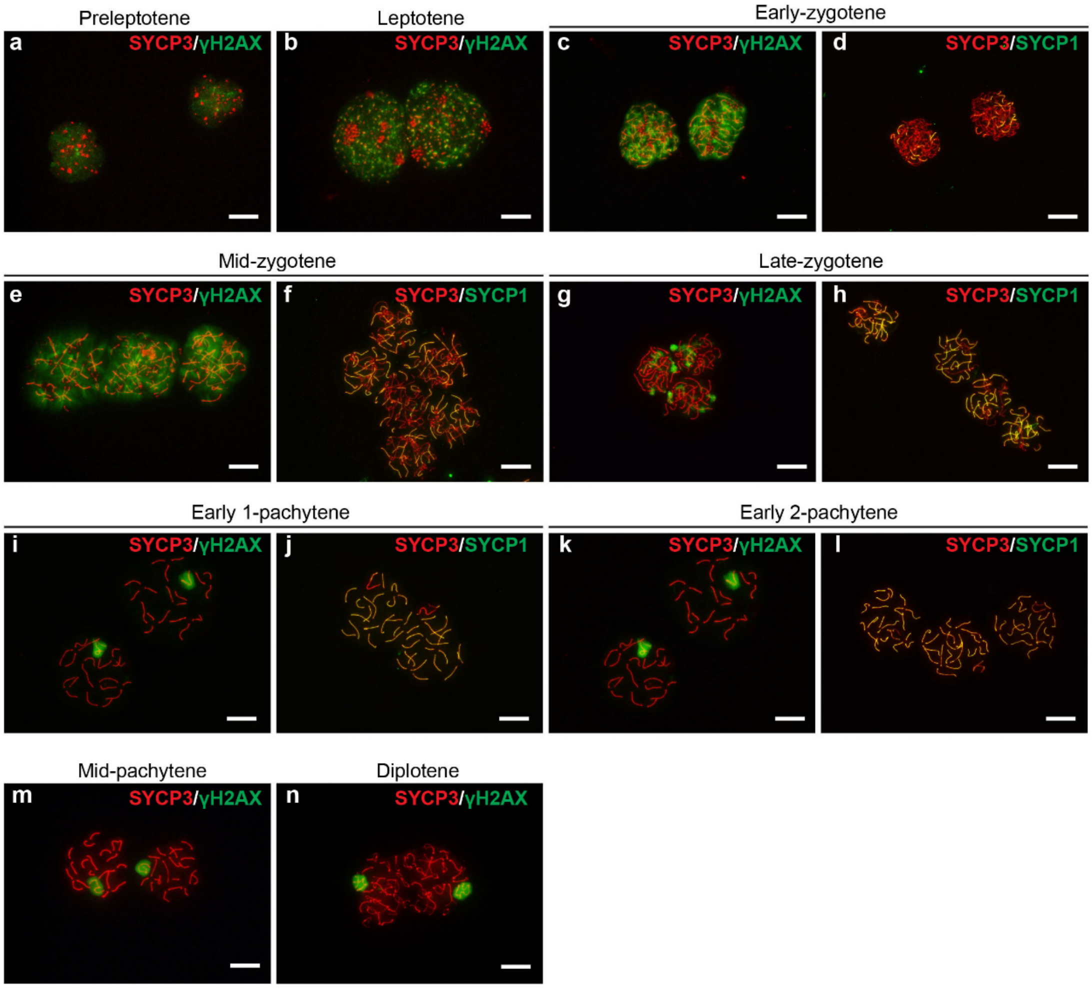

Fig. S1 Validation of the isolated spermatocytes by surface-spread chromatin immunofluorescence staining. a-n Surface-spread nuclei immunofluorescence staining for SYCP3 (red)/γH2AX (green) or SYCP3 (red)/SYCP1 (green) of the isolated spermatocytes, including preleptotene spermatocytes (**a**), leptotene spermatocytes (**b**), early-zygotene spermatocytes (**c, d**), mid-zygotene spermatocytes (**e, f**), late-zygotene spermatocytes (**g, h**), early 1-pachytene spermatocytes (**i, j**), early 2-pachytene spermatocytes (**k, l**), mid-pachytene spermatocytes (**m**), and diplotene spermatocytes (**n**). Scale bars, 40 μm.
