# Supplementary material for: Refined spatial temporal epigenomic profiling reveals intrinsic connection between PRDM9-mediated H3K4me3 and the fate of double-stranded breaks
Source: Cell Res. 2020 Feb 11;30(3):256–68. doi: 10.1038/s41422-020-0281-1 (PMC7054334; doi:10.1038/s41422-020-0281-1)
Supplement: Supplementary file 2 — Supplementary information, Figure S2 [file 41422_2020_281_MOESM2_ESM.pdf]

Supplementary information, Figure S2

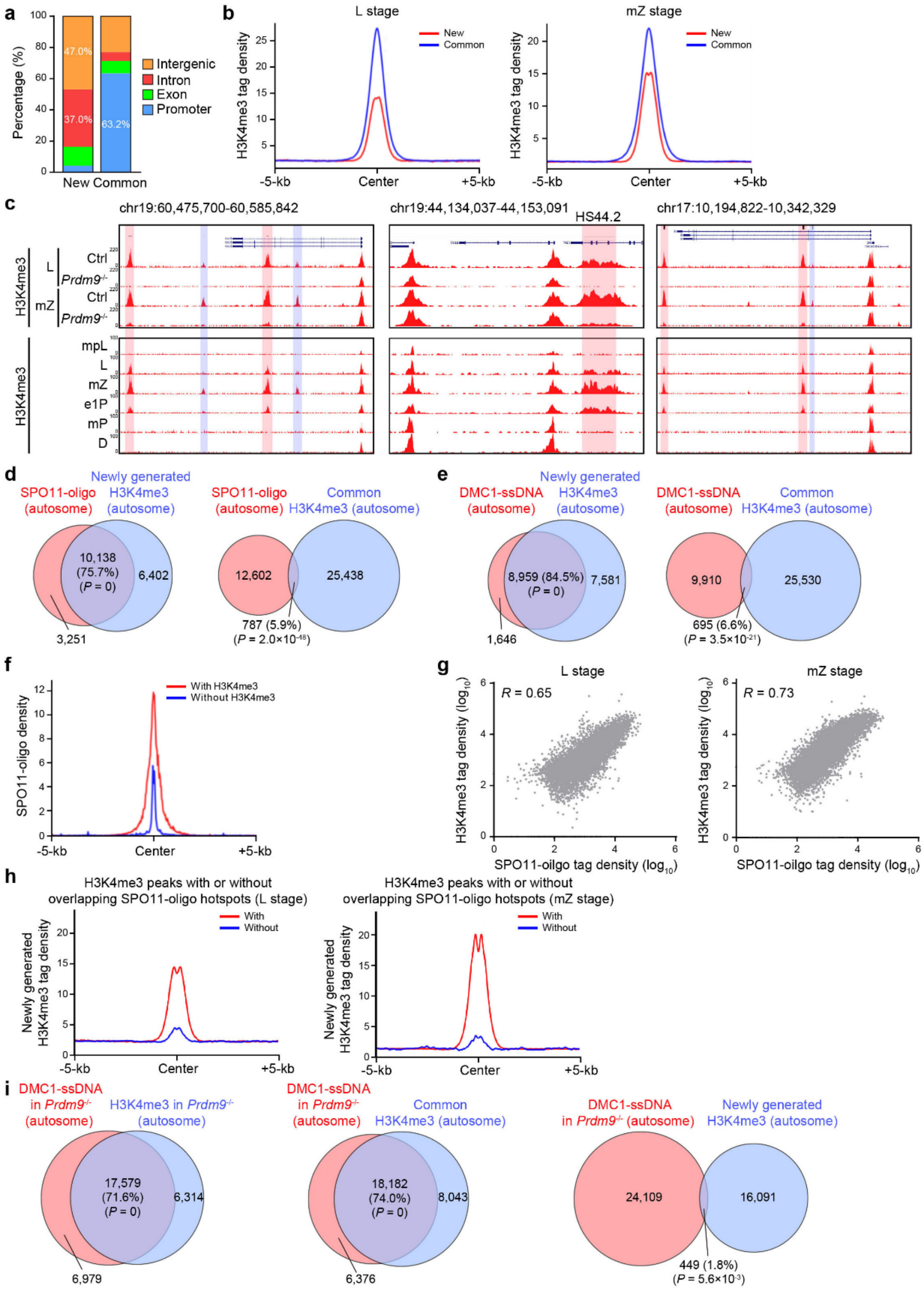

Fig. S2 Features of PRDM9-mediated H3K4me3. a Stacked bar plot showing the genomic distribution of the newly generated and common H3K4me3 peaks on different

genomic features. **b** Profile of the average H3K4me3 tag density on common and newly generated H3K4me3 peaks in leptotene (left) and mid-zygotene (right) spermatocytes. H3K4me3 tag density was calculated using H3K4me3 reads coverage with 50-bp resolution. New: newly generated H3K4me3; Common: common H3K4me3. **c** UCSC genome browser view of the PRDM9-mediated H3K4me3 peaks, the strong peaks were highlighted with red shadings and the weak peaks were highlighted with blue shadings. Ctrl: control, mpL: mid-preleptotene spermatocytes, L: leptotene spermatocytes, mZ: mid-zygotene spermatocytes, e1P: early 1-pachytene spermatocytes, mP: mid-pachytene spermatocytes, D: diplotene spermatocytes. **d** Venn diagrams showing the overlap of defined SPO11-oligo DSB hotspots with the newly generated H3K4me3 peaks (left panel), and the common H3K4me3 peaks (right panel) in mid-zygotene. **e** Venn diagrams showing the overlap of DMC1-ssDNA defined hotspots with the newly generated H3K4me3 peaks (left panel), and the common H3K4me3 peaks (right panel) in mid-zygotene. **f** Profile of the average SPO11 oligos density on SPO11-oligo defined DSB hotspots with and without newly generated H3K4me3 peaks. SPO11 oligos density was calculated using SPO11 oligos coverage with 50-bp resolution. **g** The scatter plot showing the correlation between H3K4me3 tag density and SPO11-oligos tag density on the newly generated H3K4me3 peaks. SPO11-oligo tag density and H3K4me3 tag density were summed in the 1-kb around hotspot centers. **h** Profile of the average H3K4me3 tag density on DSB hotspots with and without newly generated H3K4me3 peaks in leptotene (left) and mid-zygotene (right) spermatocytes. Note that small peak of H3K4me3 was present even for the non-overlapping set of SPO11-oligo

hotspots. **i** Venn diagrams showing the overlap of *Prdm9*$^{-/-}$ DSB hotspots with H3K4me3 in the *Prdm9*$^{-/-}$ zygotene (left panel), common H3K4me3 peaks (middle panel) and newly generated H3K4me3 peaks (right panel) in the wild-type mid-zygotene.
