# Supplementary material for: Refined spatial temporal epigenomic profiling reveals intrinsic connection between PRDM9-mediated H3K4me3 and the fate of double-stranded breaks
Source: Cell Res. 2020 Feb 11;30(3):256–68. doi: 10.1038/s41422-020-0281-1 (PMC7054334; doi:10.1038/s41422-020-0281-1)
Supplement: Supplementary file 3 — Supplementary information, Figure S3 [file 41422_2020_281_MOESM3_ESM.pdf]

Supplementary information, Figure S3

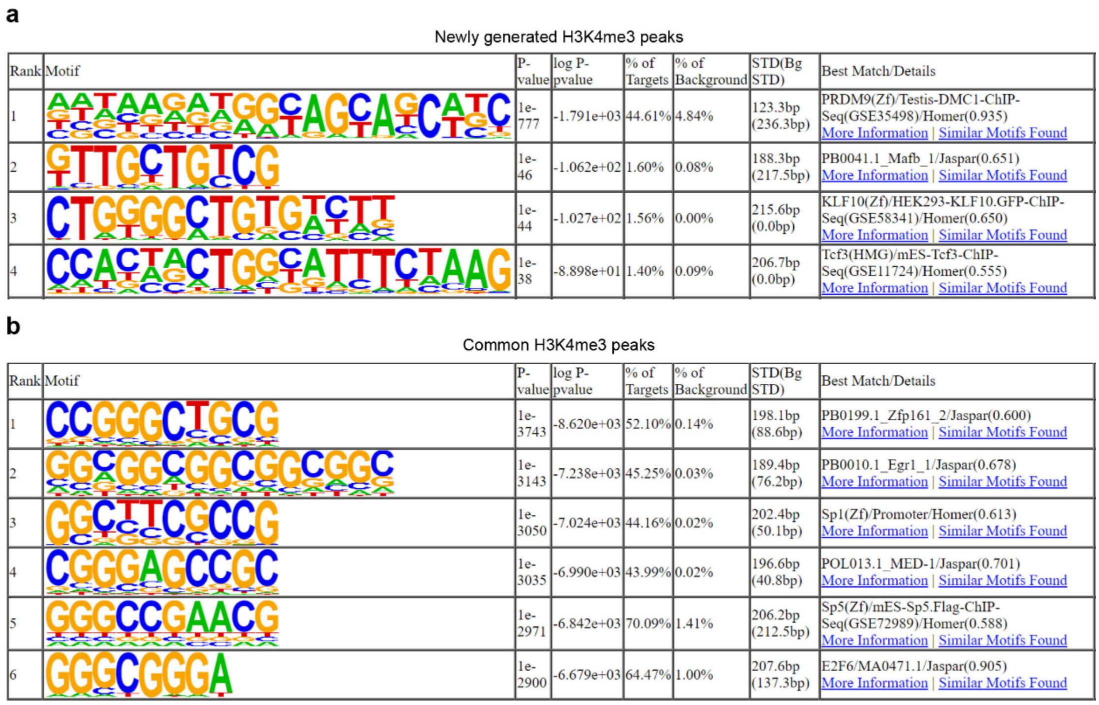

a

Newly generated H3K4me3 peaks

Rank	Motif	P-value	log P-pvalue	% of Targets	% of Background	STD(Bg STD)	Best Match/Details
1		1e-777	-1.791e+03	44.61%	4.84%	123.3bp (236.3bp)	PRDM9(Zf)/Testis-DMC1-ChIP-Seq(GSE35498)/Homer(0.935) More Information | Similar Motifs Found
2		1e-46	-1.062e+02	1.60%	0.08%	188.3bp (217.5bp)	PB0041.1_Mafb_1/Jaspar(0.651) More Information | Similar Motifs Found
3		1e-44	-1.027e+02	1.56%	0.00%	215.6bp (0.0bp)	KLF10(Zf)/HEK293-KLF10.GFP-ChIP-Seq(GSE58341)/Homer(0.650) More Information | Similar Motifs Found
4		1e-38	-8.898e+01	1.40%	0.09%	206.7bp (0.0bp)	Tcf3(HMG)/mES-Tcf3-ChIP-Seq(GSE11724)/Homer(0.555) More Information | Similar Motifs Found

b

Common H3K4me3 peaks

Rank	Motif	P-value	log P-pvalue	% of Targets	% of Background	STD(Bg STD)	Best Match/Details
1		1e-3743	-8.620e+03	52.10%	0.14%	198.1bp (88.6bp)	PB0199.1_Zfp161_2/Jaspar(0.600) More Information | Similar Motifs Found
2		1e-3143	-7.238e+03	45.25%	0.03%	189.4bp (76.2bp)	PB0010.1_Egr1_1/Jaspar(0.678) More Information | Similar Motifs Found
3		1e-3050	-7.024e+03	44.16%	0.02%	202.4bp (50.1bp)	Sp1(Zf)/Promoter/Homer(0.613) More Information | Similar Motifs Found
4		1e-3035	-6.990e+03	43.99%	0.02%	196.6bp (40.8bp)	POL013.1_MED-1/Jaspar(0.701) More Information | Similar Motifs Found
5		1e-2971	-6.842e+03	70.09%	1.41%	206.2bp (212.5bp)	Sp5(Zf)/mES-Sp5.Flag-ChIP-Seq(GSE72989)/Homer(0.588) More Information | Similar Motifs Found
6		1e-2900	-6.679e+03	64.47%	1.00%	207.6bp (137.3bp)	E2F6/MA0471.1/Jaspar(0.905) More Information | Similar Motifs Found

Fig. S3 PRDM9 DNA binding motif identified in the newly generated H3K4me3 peaks but not in common ones. a, b Identification of the predicted PRDM9 binding motif (B6 background) on the newly generated H3K4me3 peaks (**a**) and common H3K4me3 peaks (**b**).
