# Supplementary material for: Refined spatial temporal epigenomic profiling reveals intrinsic connection between PRDM9-mediated H3K4me3 and the fate of double-stranded breaks
Source: Cell Res. 2020 Feb 11;30(3):256–68. doi: 10.1038/s41422-020-0281-1 (PMC7054334; doi:10.1038/s41422-020-0281-1)
Supplement: Supplementary file 4 — Supplementary information, Figure S4 [file 41422_2020_281_MOESM4_ESM.pdf]

Supplementary information, Figure S4

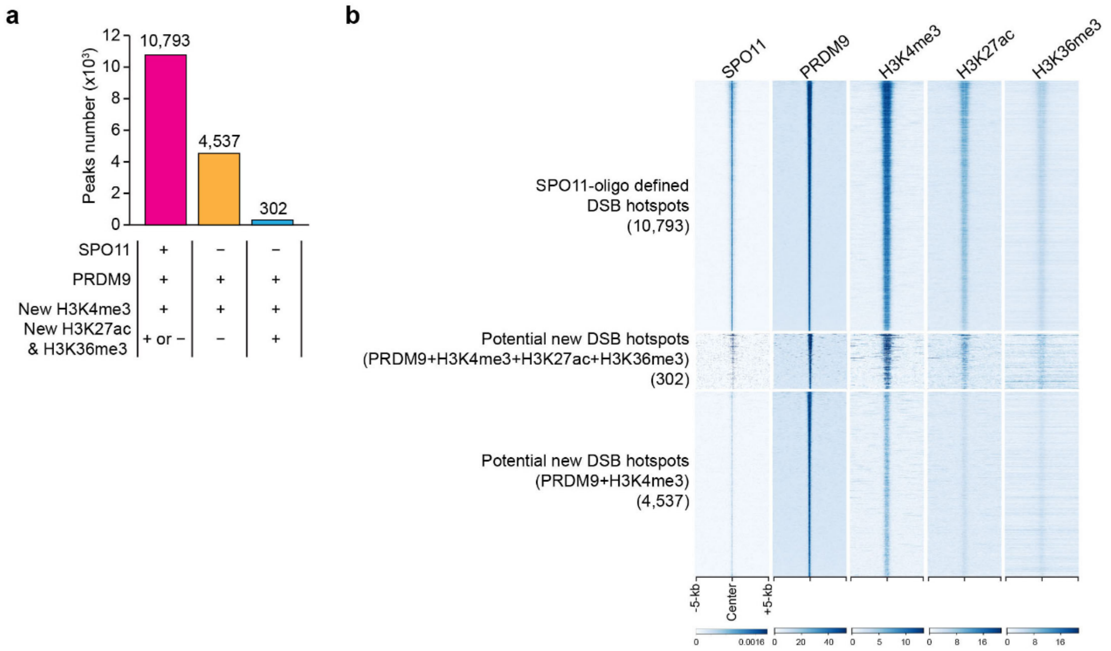

Fig. S4 Potential DSB hotspots defined by histone marks. a Histogram showing the DSB hotspots or potential DSB hotspots defined by different combinations of SPO11-oligo, Prdm9 binding sites, newly generated H3K4me3, newly generated H3K36me3 and newly generated H3K27ac. **b** Heatmaps of SPO11-oligo, Prdm9 binding sites, newly generated H3K4me3, newly generated H3K36me3 and newly generated H3K27ac on DSB hotspots or potential DSB hotspots.
