# Supplementary material for: Refined spatial temporal epigenomic profiling reveals intrinsic connection between PRDM9-mediated H3K4me3 and the fate of double-stranded breaks
Source: Cell Res. 2020 Feb 11;30(3):256–68. doi: 10.1038/s41422-020-0281-1 (PMC7054334; doi:10.1038/s41422-020-0281-1)
Supplement: Supplementary file 5 — Supplementary information, Figure S5 [file 41422_2020_281_MOESM5_ESM.pdf]

Supplementary information, Figure S5

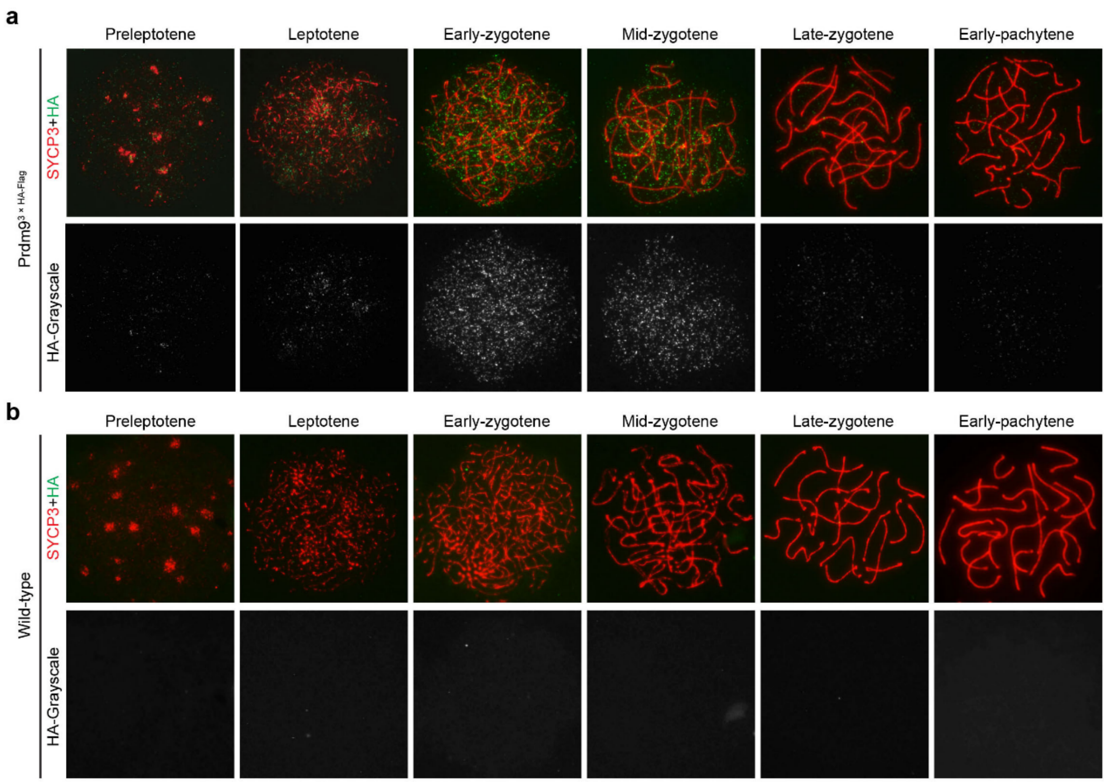

Fig. S5 Stage-specific expression of PRDM9 during meiotic prophase I. a, b Surface-spread nuclei were obtained from adult *Prdm9*-3×HA-Flag knockin (**a**) and wild-type mice for control (**b**), and immunostained with antibody to HA (green) for PRDM9, and with antibody to SYCP3 (red) for visualization of the synaptonemal complex. The grayscale of HA signals are shown for comparison of the expression levels of PRDM9.
