# Supplementary material for: Refined spatial temporal epigenomic profiling reveals intrinsic connection between PRDM9-mediated H3K4me3 and the fate of double-stranded breaks
Source: Cell Res. 2020 Feb 11;30(3):256–68. doi: 10.1038/s41422-020-0281-1 (PMC7054334; doi:10.1038/s41422-020-0281-1)
Supplement: Supplementary file 6 — Supplementary information, Figure S6 [file 41422_2020_281_MOESM6_ESM.pdf]

Supplementary information, Figure S6

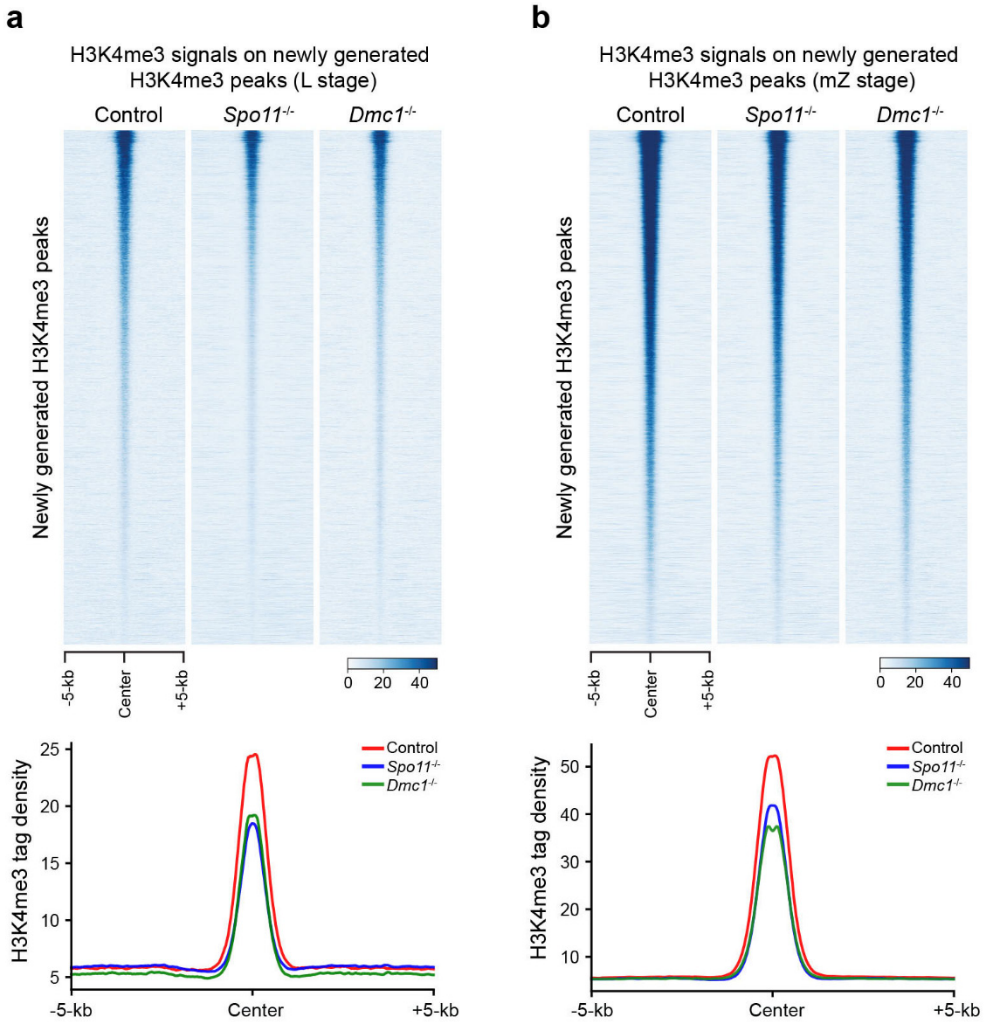

Fig. S6 H3K4me3 peaks are present at the sites of DSB hotspots in *Spo11* KO and *Dmc1* KO spermatocytes. a, b Heatmaps (top) and profiles (bottom) of normalized H3K4me3 ChIP-seq tag density on the newly generated H3K4me3 peaks in control, *Spo11*$^{-/-}$ and *Dmc1*$^{-/-}$ leptotene (**a**) and mid-zygotene (**b**) spermatocytes. Each row in heatmap represents a DSB hotspot of ± 5 kb around the center and ranked by the H3K4me3 tag density from highest to lowest. H3K4me3 tag density was calculated using H3K4me3 reads with 50-bp resolution.
