# Supplementary material for: Refined spatial temporal epigenomic profiling reveals intrinsic connection between PRDM9-mediated H3K4me3 and the fate of double-stranded breaks
Source: Cell Res. 2020 Feb 11;30(3):256–68. doi: 10.1038/s41422-020-0281-1 (PMC7054334; doi:10.1038/s41422-020-0281-1)
Supplement: Supplementary file 7 — Supplementary information, Figure S7 [file 41422_2020_281_MOESM7_ESM.pdf]

Supplementary information, Figure S7

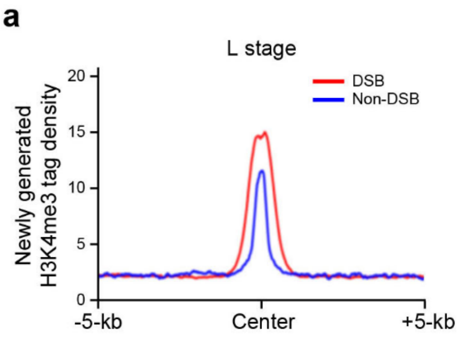

Fig. S7 The strength of hotspot-associated H3K4me3 is stronger than non-hotspot H3K4me3. a, b Profile of the average H3K4me3 tag density on hotspot-associated H3K4me3 (DSB) peaks and non-hotspots H3K4me3 (non-DSB) peaks in leptotene (**a**) and mid-zygotene (**b**) spermatocytes. H3K4me3 tag density was calculated using H3K4me3 ChIP-seq reads with 50-bp resolution.
