# Supplementary material for: Refined spatial temporal epigenomic profiling reveals intrinsic connection between PRDM9-mediated H3K4me3 and the fate of double-stranded breaks
Source: Cell Res. 2020 Feb 11;30(3):256–68. doi: 10.1038/s41422-020-0281-1 (PMC7054334; doi:10.1038/s41422-020-0281-1)
Supplement: Supplementary file 8 — Supplementary information, Figure S8 [file 41422_2020_281_MOESM8_ESM.pdf]

Supplementary information, Figure S8

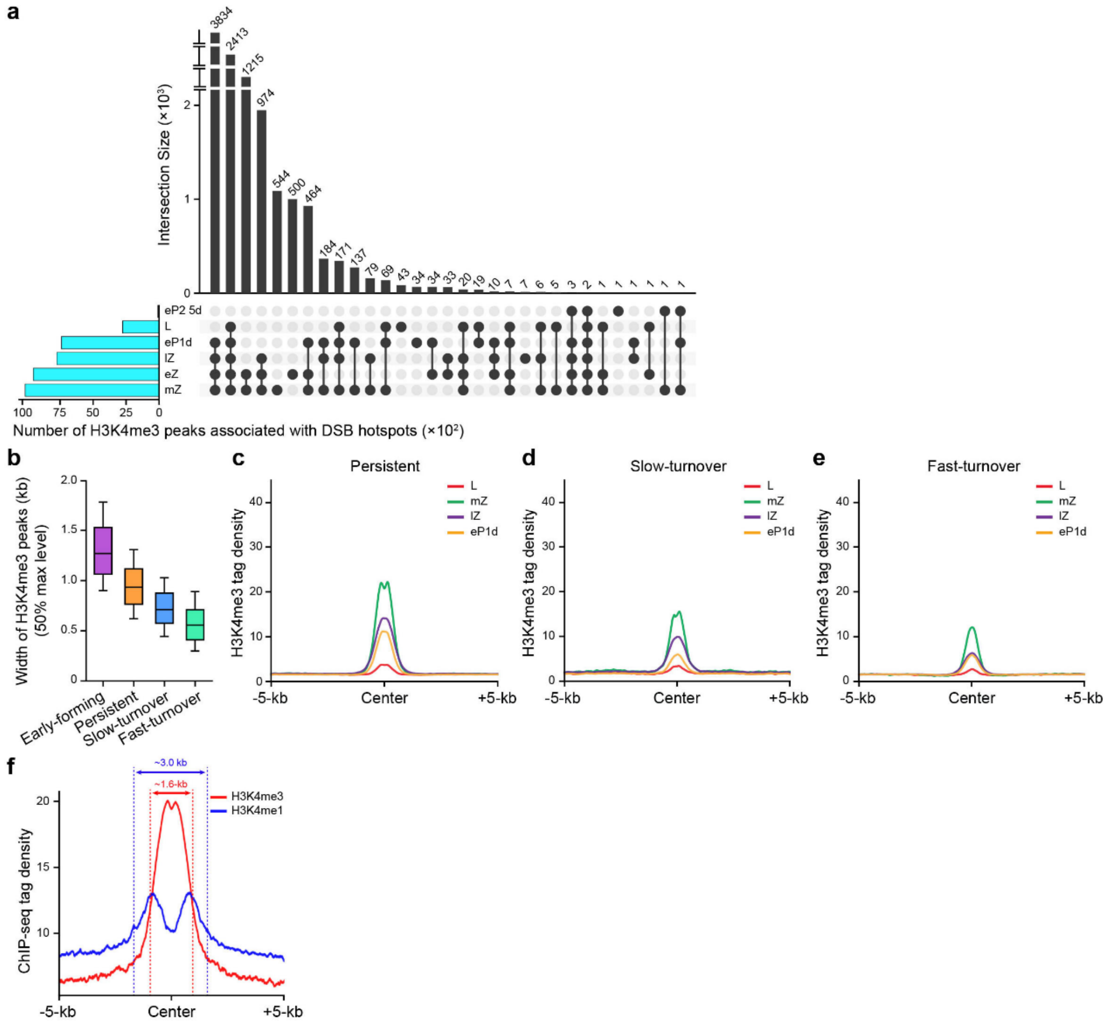

Fig. S8 The dynamics of hotspot-associated H3K4me3 peaks. a Point vennplot diagram showing the overlap of hotspot-associated H3K4me3 peaks among leptotene, early-zygotene, mid-zygotene, late-zygotene, early 1-pachytene spermatocytes and early 2-pachytene spermatocytes. **b** Boxplot showing the width of the four subtypes of hotspot-associated H3K4me3 peaks at mid-zygotene, which are normalized by the width at 50% max level. **c-e** Profile of the averaged H3K4me3 tag density on persistent (**c**), slow-turnover (**d**) or fast-turnover (**e**) hotspot-associated H3K4me3 peaks in

leptotene, mid-zygotene, late-zygotene, and early 1-pachytene. H3K4me3 tag density was calculated using H3K4me3 ChIP-seq reads coverage with 50-bp resolution. **f** Profile of the average H3K4me1 and H3K4me3 tag density on the top 3000 DSB hotspots defined using SPO11-oligo in mid-zygotene spermatocytes. H3K4me3 tag density was calculated using H3K4me1 or H3K4me3 ChIP-seq reads with 50-bp resolution, the width at the 50% max heights of H3K4me3 and H3K4me1 peaks were shown.
