# Supplementary material for: Refined spatial temporal epigenomic profiling reveals intrinsic connection between PRDM9-mediated H3K4me3 and the fate of double-stranded breaks
Source: Cell Res. 2020 Feb 11;30(3):256–68. doi: 10.1038/s41422-020-0281-1 (PMC7054334; doi:10.1038/s41422-020-0281-1)
Supplement: Supplementary file 9 — Supplementary information, Figure S9 [file 41422_2020_281_MOESM9_ESM.pdf]

Supplementary information, Figure S9

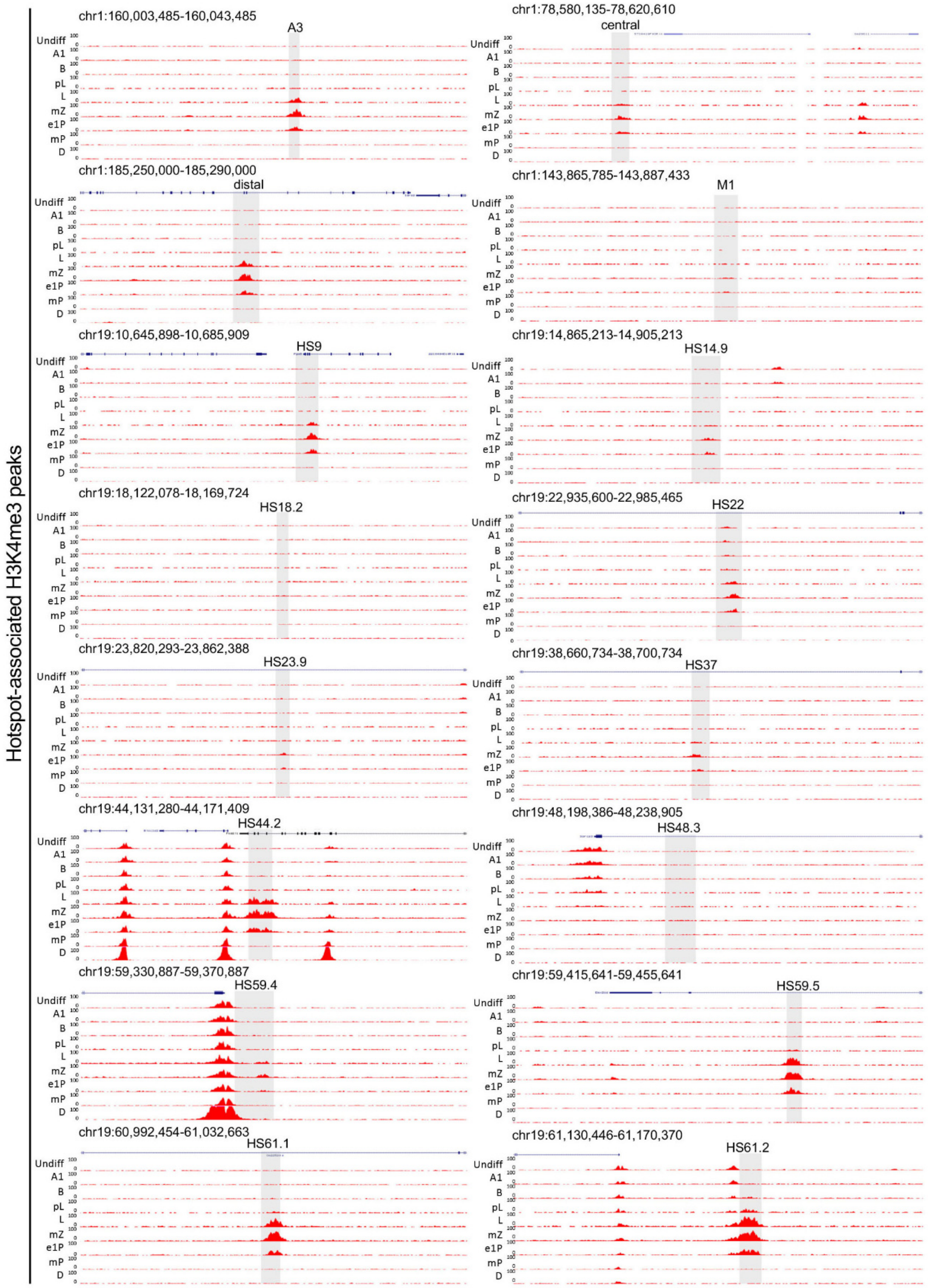

Fig. S9 14 of 16 known crossover hotspots contain hotspot-associated H3K4me3 that are present from leptotene to early 1-pachytene. UCSC genome browser view

of H3K4me3 reads coverage on 16 known crossover hotspots in different stages of spermatogonia, and various substages of meiotic prophase I cells. The positions of crossover hotspots are highlighted in the light black shading. Undiff: undifferentiated spermatogonia, A1: type A1 spermatogonia, B: type B spermatogonia, pL: mid-preleptotene, L: leptotene, mZ: mid-zygotene, e1P: early 1-pachytene, mP: mid-pachytene, D: diplotene.
