# Supplementary material for: Refined spatial temporal epigenomic profiling reveals intrinsic connection between PRDM9-mediated H3K4me3 and the fate of double-stranded breaks
Source: Cell Res. 2020 Feb 11;30(3):256–68. doi: 10.1038/s41422-020-0281-1 (PMC7054334; doi:10.1038/s41422-020-0281-1)
Supplement: Supplementary file 10 — Supplementary information, Figure S10 [file 41422_2020_281_MOESM10_ESM.pdf]

Supplementary information, Figure S10

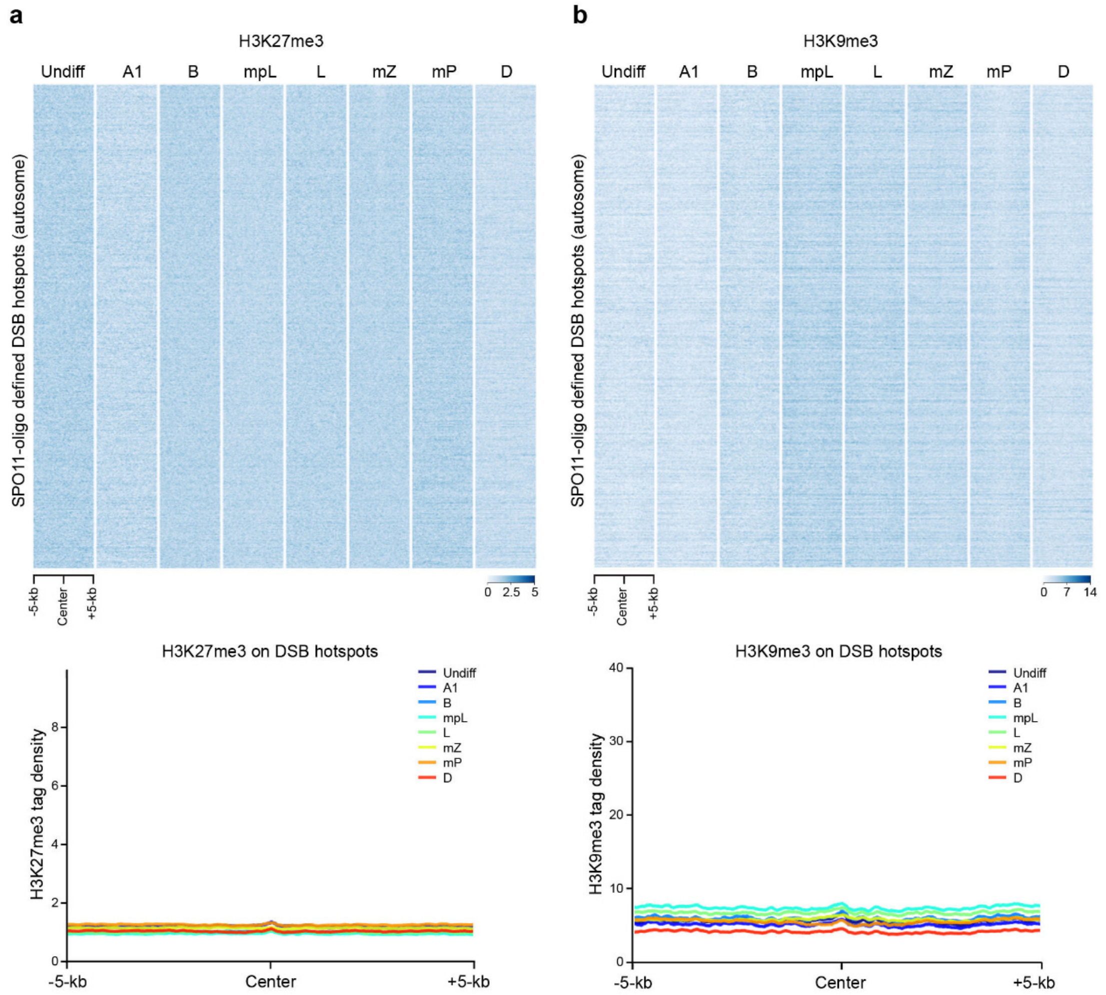

Fig. S10 No significant enrichment of H3K27me3 and H3K9me3 on DSB hotspots.

a, b Heatmaps (top) and profiles (bottom) of H3K27me3 (**a**) and H3K9me3 (**b**) tag density on the SPO11-oligo defined DSB hotspots in spermatogenic cells. Each row in heatmap represents a DSB hotspot of ± 5 kb around the center and ranked by SPO11-oligo density from highest and lowest. Color indicates H3K4me3 tag density. Average ChIP-seq tag density was calculated using ChIP-seq reads with 50-bp resolution. Undiff: undifferentiated spermatogonia, A1: type A1 spermatogonia, B: type B spermatogonia, mpL: mid-preleptotene, L: leptotene, mZ: mid-zygotene, mP: mid-pachytene, D: diplotene.
