# Supplementary material for: Refined spatial temporal epigenomic profiling reveals intrinsic connection between PRDM9-mediated H3K4me3 and the fate of double-stranded breaks
Source: Cell Res. 2020 Feb 11;30(3):256–68. doi: 10.1038/s41422-020-0281-1 (PMC7054334; doi:10.1038/s41422-020-0281-1)
Supplement: Supplementary file 11 — Supplementary information, Figure S11 [file 41422_2020_281_MOESM11_ESM.pdf]

Supplementary information, Figure S11

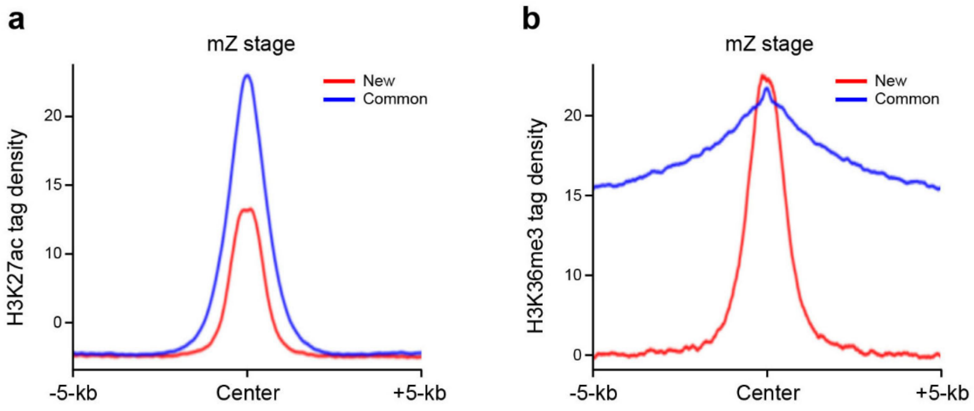

Fig. S11 The average strengths of the newly generated H3K27ac and H3K36me3 are much weaker than those of the common ones. a Profile of the average H3K27ac tag density on newly generated H3K27ac peaks and common H3K27ac peaks in mid-zygotene spermatocytes. H3K27ac tag density was calculated using H3K27ac reads coverage with 50-bp resolution. **b** Profile of the average H3K36me3 tag density on newly generated H3K36me3 peaks and common H3K36me3 peaks in mid-zygotene spermatocytes. H3K36me3 tag density was calculated using H3K36me3 reads coverage with 50-bp resolution.
