# Supplementary material for: Refined spatial temporal epigenomic profiling reveals intrinsic connection between PRDM9-mediated H3K4me3 and the fate of double-stranded breaks
Source: Cell Res. 2020 Feb 11;30(3):256–68. doi: 10.1038/s41422-020-0281-1 (PMC7054334; doi:10.1038/s41422-020-0281-1)
Supplement: Supplementary file 12 — Supplementary information, Figure S12 [file 41422_2020_281_MOESM12_ESM.pdf]

Supplementary information, Figure S12

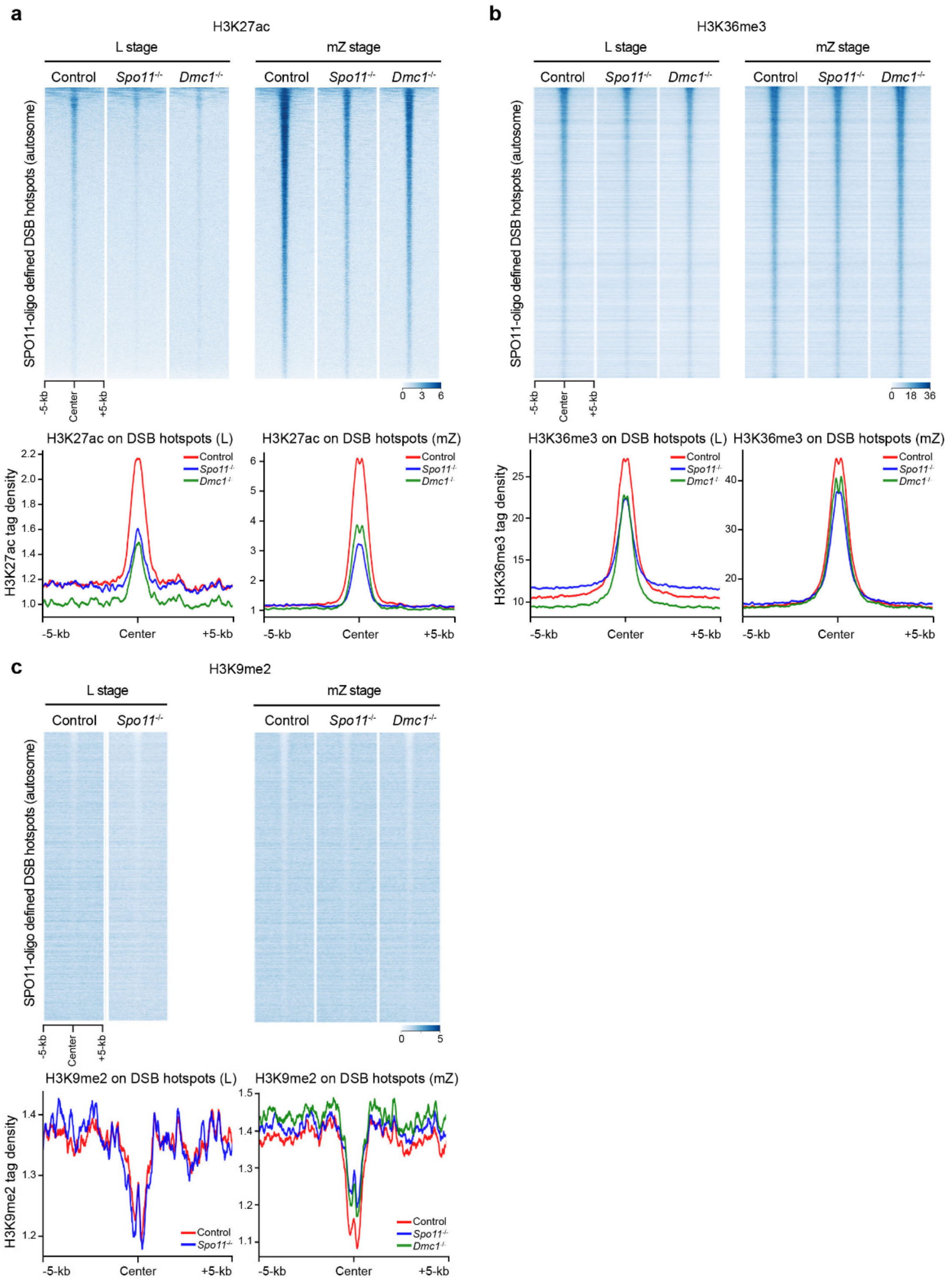

Fig. S12 H3K27ac and H3K36me3 peaks, as well as H3K9me2 dips are present at the sites of DSB hotspots in *Spo11*$^{-/-}$ and *Dmc1*$^{-/-}$ spermatocytes. a Heatmaps (top)

and profiles (bottom) of H3K27ac tag density on the SPO11-oligo defined DSB hotspots in control, *Spo11*$^{-/-}$ and *Dmc1*$^{-/-}$ leptotene (left) or mid-zygotene (right) spermatocytes. Each row in heatmaps represents a DSB hotspot of ± 5 kb around the center and ranked by SPO11-oligo density from highest and lowest. Color indicates ChIP-seq tag density. Average ChIP-seq tag density was calculated using ChIP-seq reads coverage with 50-bp resolution. **b** Heatmaps (top) and profiles (bottom) of H3K36me3 tag density on the SPO11-oligo defined DSB hotspots in control, *Spo11*$^{-/-}$ and *Dmc1*$^{-/-}$ leptotene (left) or mid-zygotene (right) spermatocytes. Each row in heatmaps represents a DSB hotspot of ± 5 kb around the center and ranked by SPO11-oligo density from highest and lowest. Color indicates ChIP-seq tag density. Average ChIP-seq tag density was calculated using ChIP-seq reads coverage with 50-bp resolution. **c** Heatmaps (top) and profiles (bottom) of H3K9me2 tag density on the SPO11-oligo defined DSB hotspots in control, *Spo11*$^{-/-}$ and *Dmc1*$^{-/-}$ leptotene (left) or mid-zygotene (right) spermatocytes. Each row in heatmaps represents a DSB hotspot region of ± 5 kb around the center and ranked by SPO11-oligo density from highest and lowest. Color indicates ChIP-seq tag density. Average ChIP-seq tag density was calculated using ChIP-seq reads coverage with 50-bp resolution.
