# Supplementary material for: Refined spatial temporal epigenomic profiling reveals intrinsic connection between PRDM9-mediated H3K4me3 and the fate of double-stranded breaks
Source: Cell Res. 2020 Feb 11;30(3):256–68. doi: 10.1038/s41422-020-0281-1 (PMC7054334; doi:10.1038/s41422-020-0281-1)
Supplement: Supplementary file 13 — Supplementary information, Figure S13 [file 41422_2020_281_MOESM13_ESM.pdf]

Supplementary information, Figure S13

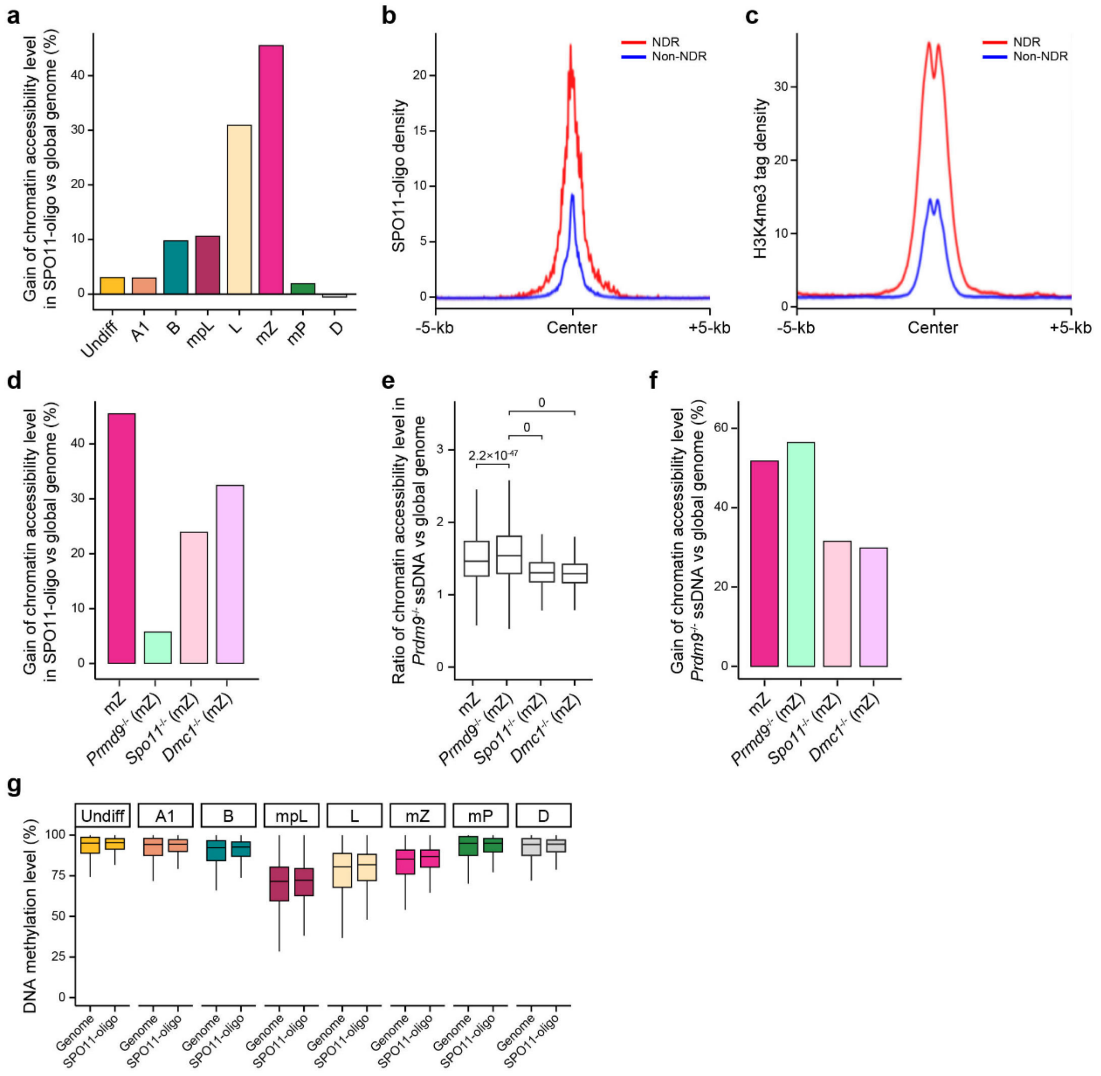

Fig. S13 Chromatin accessibility of the hotspots are positively correlated with DSB hotspots and H3K4me3 signals. a Histogram showing the gain of chromatin accessibility level (chromatin accessibility level in SPO11-oligo defined DSB hotspots versus that in the whole genome) in different stages of spermatogenic cells. Only regions with at least three GCH sites were considered. **b** Profile of the average SPO11-oligo density on SPO11-oligo defined DSB hotspots with NDRs and without NDRs. **c** Profile of the average H3K4me3 density on SPO11-oligo defined DSB hotspots with

NDRs and without NDRs. **d** Histogram showing the gain of chromatin accessibility level (chromatin accessibility level in SPO11-oligo defined DSB hotspots versus that in the whole genome) in the wild-type, *Prdm9*$^{-/-}$, *Spo11*$^{-/-}$ and *Dmc1*$^{-/-}$ mid-zygotene spermatocytes. Only regions with at least three GCH sites were considered. **e** Boxplot showing the ratio of chromatin accessibility level (chromatin accessibility level in *Prdm9*$^{-/-}$ DMC1-ssDNA defined DSB hotspots versus that in the whole genome) in the wild-type, *Prdm9*$^{-/-}$, *Spo11*$^{-/-}$, and *Dmc1*$^{-/-}$ mid-zygotene spermatocytes. Only regions with at least three GCH sites were considered. **f** Histogram showing the gain of chromatin accessibility level (chromatin accessibility level in *Prdm9*$^{-/-}$ DMC1-ssDNA defined DSB hotspots versus that in the whole genome) in the wild-type, *Prdm9*$^{-/-}$, *Spo11*$^{-/-}$, and *Dmc1*$^{-/-}$ mid-zygotene spermatocytes. Only regions with at least three GCH sites were considered. **g** DNA methylation level in SPO11-oligo defined DSB hotspots and the whole genome. DSB hotspots were extended as center ± 2 kb and the whole genome was separated into several tiles with 1-kb size to be calculated the mean DNA methylation. Only regions with at least three WCG sites were considered. Undiff: undifferentiated spermatogonia, A1: type A1 spermatogonia, B: type B spermatogonia, mpL: mid-preleptotene, L: leptotene, mZ: mid-zygotene, mP: mid-pachytene, D: diplotene.
