# Supplementary material for: Refined spatial temporal epigenomic profiling reveals intrinsic connection between PRDM9-mediated H3K4me3 and the fate of double-stranded breaks
Source: Cell Res. 2020 Feb 11;30(3):256–68. doi: 10.1038/s41422-020-0281-1 (PMC7054334; doi:10.1038/s41422-020-0281-1)
Supplement: Supplementary file 14 — Supplementary information, Figure S14 [file 41422_2020_281_MOESM14_ESM.pdf]

Supplementary information, Figure S14

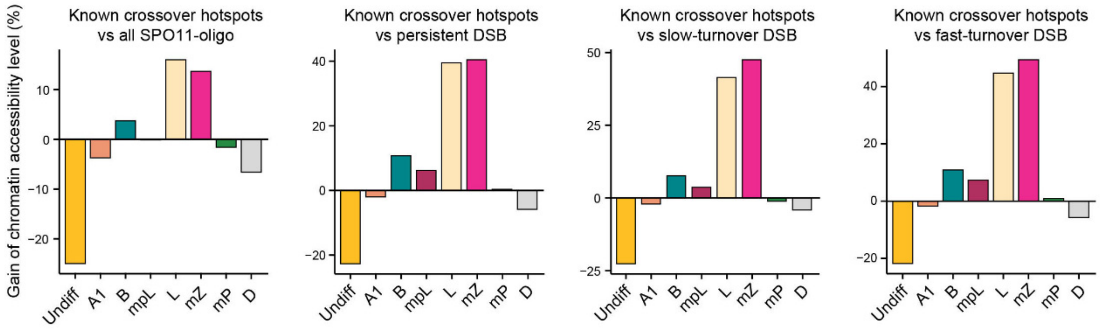

Fig. S14 Chromatin states around the 16 well-known CO hotspots and general DSB hotspots. Histogram showing the gain of chromatin accessibility level (chromatin accessibility level in known crossover hotspots versus that in general SPO11-oligo defined DSB hotspots) in different stages of spermatogenic cells. Undiff: undifferentiated spermatogonia, A1: type A1 spermatogonia, B: type B spermatogonia, mpL: mid-preleptotene, L: leptotene, mZ: mid-zygotene, mP: mid-pachytene, D: diplotene.
