# Supplementary material for: Refined spatial temporal epigenomic profiling reveals intrinsic connection between PRDM9-mediated H3K4me3 and the fate of double-stranded breaks
Source: Cell Res. 2020 Feb 11;30(3):256–68. doi: 10.1038/s41422-020-0281-1 (PMC7054334; doi:10.1038/s41422-020-0281-1)
Supplement: Supplementary file 15 — Supplementary information, Figure S15 [file 41422_2020_281_MOESM15_ESM.pdf]

Supplementary information, Figure S15

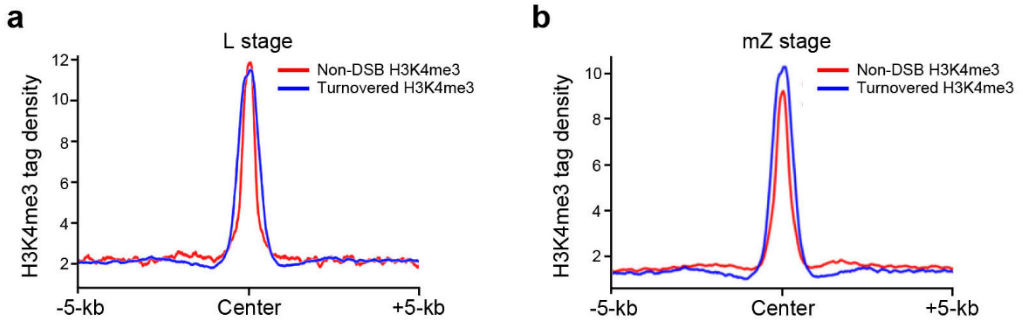

Fig. S15 The strength of non-hotspot PRDM9-mediated H3K4me3 in both leptotene and zygotene is similar to that of fast- and slow-turnover H3K4me3. a, b Profile of the average H3K4me3 tag density on non-hotspot H3K4me3 (non-DSB H3K4me3) peaks and fast- and slow-turnover H3K4me3 (turnovered H3K4me3) peaks in leptotene stage (**a**) and mid-zygotnene stage (**b**). H3K4me3 tag density was calculated using H3K4me3 read coverage with 50-bp resolution.
